# Dogs (*Canis familiaris*) as Sentinels for Human Infectious Disease and Application to Canadian Populations: A Systematic Review

**DOI:** 10.3390/vetsci5040083

**Published:** 2018-09-21

**Authors:** Natasha H. Bowser, Neil E. Anderson

**Affiliations:** The Royal (Dick) School of Veterinary Studies and the Roslin Institute, University of Edinburgh, Roslin EH25 9RG, UK; nhbowser@gmail.com

**Keywords:** One Health, dogs, sentinel surveillance, infectious disease, emerging disease, zoonosis

## Abstract

In a world where climate change, vector expansion, human activity, and pathogen dispersal do not respect boundaries, the human–animal–pathogen interface has become less defined. Consequently, a One Health approach to disease surveillance and control has generated much interest across several disciplines. This systematic review evaluates current global research on the use of domestic dogs as sentinels for human infectious disease, and critically appraises how this may be applied within Canada. Results highlighted a bias in research from high- and middle-income-economy countries, with 35% of the studies describing data from the Latin America/Caribbean region, 25% from North America, and 11% from the European/Central Asia region. Bacteria were the most studied type of infectious agent, followed by protozoa, viruses, helminths, and fungi. Only six out of 142 studies described disease in Canada: four researched a variety of pathogens within Indigenous communities, one researched *Borrelia burgdorferi* in British Columbia, and one researched arboviruses in Quebec. Results from this review suggest that dogs could provide excellent sentinels for certain infectious-disease pathogens in Canada, yet are currently overlooked. Further research into the use of dog-sentinel surveillance is specifically recommended for California serogroup viruses, Chikungunya virus, West Nile virus, Lyme borreliosis, *Rickettsia* spp., *Ehrlichia* spp., and *Dirofilaria immitis*.

## 1. Introduction

### 1.1. One Health

The One Health Commission defines One Health as “the collaborative effort of multiple health-science professions, together with their related disciplines and institutions—working locally, nationally, and globally—to attain optimal health for people, domestic animals, wildlife, plants, and our environment” [1]. The focus of One Health research and activities has largely stemmed from zoonotic disease activities and prediction of pathogen emergence at the animal–human interface, such as avian influenza and severe acute respiratory syndrome (SARS); however, the multifaceted and wide-approach scope of this concept extends to “megaconcerns”, such as food security, food safety, antimicrobial resistance, and climate change, as well as the human–animal bond and socioeconomic fields [2,3]. While the One Health concept has attracted interest across veterinary, medical, conservation, and socioeconomic domains, concerns have been raised over the lack of governance in global health issues, and the difficulties of breaking down the siloed approach to health and translating ideas into action, particularly in developing countries [4,5,6]. Sentinel surveillance can provide a useful framework for enhancing collaboration across sectors and reducing these so-called “silos”.

An emerging pathogen has been defined by Woolhouse as “an infectious agent whose incidence is increasing following its first introduction into a new host population” [7]; however, the terms “emerging” and “re-emerging” are often used in a more comprehensive manner, as highlighted by Millar and Moore [8]. It has been estimated that 61% of all known human pathogens are zoonotic [9], and that 60–75% of emerging pathogens are, or were originally, zoonotic [9,10]. Overall, viral and protozoal pathogens have been considered more likely to emerge, and helminths less likely, regardless of zoonotic potential [9]. However, newer research suggests bacteria and rickettsia are responsible for 54% of emerging infectious diseases [10]. A pathogen’s ability to infect multiple species has also been associated with an increased risk of emergence in humans and livestock [11], and it has been suggested that, given the diversity of emerging and re-emerging pathogens, broadly targeted surveillance to include nonhuman populations may be most effective and useful for monitoring infectious-disease trends [12]. It is within this context of infectious disease and One Health that the current review is framed.

### 1.2. Sentinel Surveillance

Sentinel surveillance involves surveillance of targeted subpopulation(s), which may improve both detection of disease and cost effectiveness [13]. In simple terms, a sentinel may be defined as “an indicator of the presence of disease” [14]. Animals may be used as sentinels for various health risks, and a classic example would be ‘the canary in a coal mine’ [15]. This illustrates sentinel surveillance for an environmental hazard, but animals may also serve as sentinels for food-related hazards, infectious disease, and bioterrorism [15]. In terms of infectious disease, animal sentinels may be used to detect pathogens or disease outbreaks in a new area, monitor changes in prevalence or incidence, or track expansion of a pathogen over time and space. They may also be used to test a hypothesis related to a pathogen’s epidemiology, to evaluate effectiveness of disease-control interventions, or to assess risk factors to a population [15,16]. The ideal sentinel would be susceptible to but also survive infection, and develop a detectable and measurable response, whether clinical or immunological. Increased exposure and/or susceptibility compared to the target population could provide earlier detection and disease-control responses. Furthermore, the ideal sentinel would pose no risk to people in contact with them (i.e., minimal risk of zoonotic transmission), and not contribute to propagation of the disease through amplification, infectious viremia, and infection of vectors [15,17]. Sentinels are therefore not appropriate for detection of unknown pathogens as it is not possible to detect and measure an unknown response to a pathogen.

Despite promising research into the use of animal sentinels, it has been argued that they remain underutilized, particularly regarding infectious disease. This has been attributed, at least in part, to a need for greater data sharing, integration and cooperation between the human and animal medical professions, more robust study methods, and the need for standardized criteria to evaluate the potential of animals as sentinels [16,18]. Examples of animal sentinel-surveillance programs include the monitoring of wild birds for the West Nile virus and avian influenza by the Canadian Wildlife Health Cooperative, in collaboration with provincial/territorial governments and federal agencies. A conceptual framework to evaluate animal populations as sentinels for infectious-disease surveillance has been described [16], and the Yale Canary Database compiles peer-reviewed research related to animal sentinel surveillance of human health hazards [19].

### 1.3. Dogs as Sentinels

Several positive attributes of the domestic dog (*Canis familiaris*) have been described in the context of utilizing them as sentinels for human disease. In many countries they are ubiquitous, with free-roaming and scavenging lifestyles, thus exposing them to multiple pathogens and making them an ideal “sampling tool” [20]. In addition, as with other scavengers, pathogens may bioaccumulate in the dog after eating infected prey species, thereby enabling them to represent pathogens present in wider populations at a low prevalence or in populations difficult to sample. Using dogs to study pathogens in wildlife species may be preferable not only because of increased efficiency and ease of sampling, but also to prevent sampling and observation from affecting the study system [16]. However, free-roaming dogs sharing a geographic range with canine and non-canine wildlife species also provide a bridge between humans and wildlife, thus potentially serving as a source of human infection [21]. Such populations have been implicated in disease outbreaks in wildlife globally [22]. Thus, dogs may act as sentinels in certain situations, but also as sources of infection.

In other environments, dogs may live in close proximity to their owners, often sleeping within the same room and travelling together, and therefore having shared exposure to household and recreational risk factors, whereby the health of the dog may mirror that of their owner [15]. Most research into dog sentinel-surveillance has been related to environmental health risks, and the limitations and barriers to the use of such data for making human healthcare decisions have been well-described [18,23]. These include a lack of case-control and cohort studies, appropriate sample sizes, and relevance to human health in terms of shared exposures, outcomes, and susceptibilities between sentinel animals and humans. One example where dogs acted as sentinels for potential human food contamination is when, following reports of renal failure in dogs and cats, recalls of pet food containing melamine-contaminated wheat gluten led to identification of the contaminated gluten in the food of pigs and chickens destined for human consumption [24].

There were two objectives for the review presented in this manuscript. Firstly, to assess existing research related to the use of dogs as sentinels for human infectious disease globally. Secondly, to critically evaluate which pathogens may be most suited to sentinel surveillance in Canada using dogs. It is hoped that the results will guide more focused research on specific pathogens, which may lead to surveillance activities aimed at detecting disease before human cases occur and involving collaboration between animal and human health sectors.

## 2. Materials and Methods

A systematic literature review of papers demonstrating or suggesting use of dogs as sentinels for human infectious disease was undertaken in line with PRISMA guidelines [25], incorporating various study types, to “scope out” the current knowledge base around the use of dogs as sentinels. Due to the broad, scoping nature of the research questions, assessing study design and quality were not objectives. The research questions were:

1. Globally, what research has been undertaken related to the current use or suggestion of dogs as sentinels for human infectious disease?
2. How much of this research is related to Canadian populations, and which research could be applied to Canadian populations?

Five databases were searched: Web of Science, PubMed, Global Health (CABI), CAB Abstracts, and Google Scholar. Google Scholar was used to search and scan for additional publications not found in the other databases, using the same search terms (given below). The limits applied to the search were that they be available in English (in order for the person performing the review to be able to read and thoroughly understand the context), and that the search terms be present in the title and abstract, or the whole article if the option to select title and abstract was not available. There were no limits to the type of study or the year of publication. Grey literature, such as government documents, reports, and working papers, was excluded. Reference lists of publications were scanned for additional relevant publications. The final search was conducted on 1 December 2017.

The search terms used are shown in Table 1. Search terms for “dog”, “sentinel”, and “disease” were combined using the ‘AND’ Boolean operator.

Inclusion criteria specified that the article must pertain to an infectious disease of public health significance, relate to dogs (*Canis familiaris*) regardless of type, be original research, demonstrate or suggest the use of dogs as sentinels, describe natural infection, and be available in English. Exclusion criteria were: not pertaining to dogs specifically as sentinels (therefore, dogs noted only as carriers or reservoirs were excluded), grey literature, full paper unavailable, and unavailable in English.

**Figure 1:**
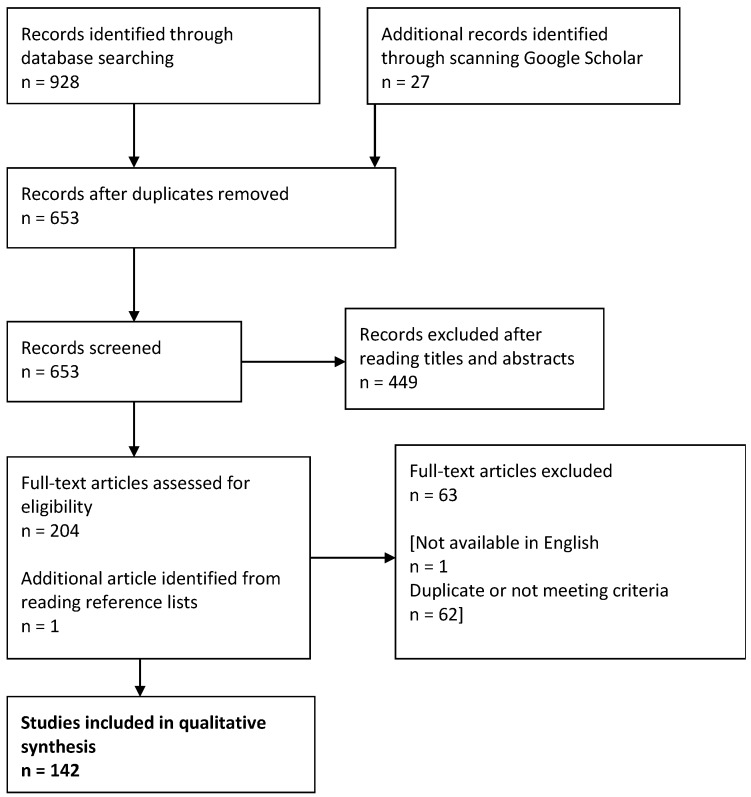
Process of refining results from the systematic review.

Figure 1 shows the process of refining the literature. In total, 142 results were obtained (full list accessible in Supplementary Materials, File S1). Information relevant to the research questions was extracted into a data-capture form (an example of which is accessible in Supplementary Materials, Table S1) and imported into a Microsoft Excel spreadsheet for analysis.

## 3. Review of the Global Literature

### 3.1. Description of Obtained Results

141/142 results were from peer-reviewed journals. The most common study method was serosurvey of dogs, in which dogs were used to estimate seroprevalences, employed in 108 (76.1%) of the studies. Study methods were varied, including serosurveys of both dogs and humans, comparisons of dog seroprevalence with previously documented human incidence, seroprevalence during endemic activity or after a case or epidemic, seroconversion studies, measuring effectiveness of an intervention, evaluating absence of disease, preparing risk assessment, case reports/series, geospatial/temporal analysis, utilizing dogs as natural-parasite models, parasite analysis, and fecal and tissue analysis. A wide variety of canine populations were utilized in the identified publications. Categories of dog populations included owned domestic dogs, rural working dogs, stray and shelter dogs, and working military or police dogs, among others.

**Figure 2:**
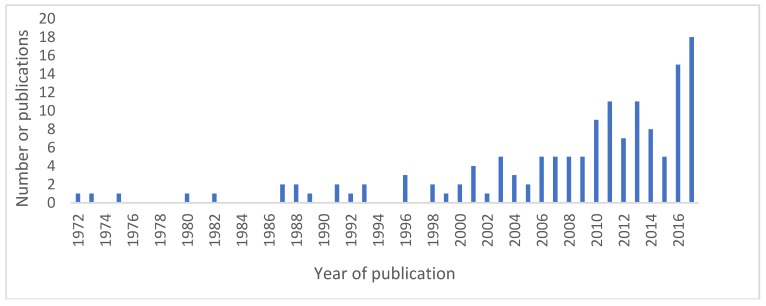
Number of publications over time.

Publication dates ranged from 1972 to 2017, with an increasing number of papers published over time, as shown in Figure 2. This may reflect the increased awareness and interest in animal–human inter-relatedness, central to the One Health concept, and greater recognition of the value of sentinel surveillance.

Countries of data collection were grouped according to the World Bank global geographic regions [26]. Of the 142 results, 49 (35%) collected data from Latin America and the Caribbean, 36 (25%) from North America, 24 (17%) from Europe and Central Asia, 16 (11%) from East Asia and the Pacific, eight (6%) from the Middle East and North Africa, and eight (6%) from Sub-Saharan Africa. One study collected data from multiple countries across different regions.

When analyzed by World Bank global regions according to income (GDP), it was unsurprising to find that 69/142 (49%) results collected data from high-income economies, 57/142 (40%), from upper-middle-income economies, 10/142 (7%) from lower-middle-income economies, and five out of 142 (4%) from low-income economies. This bias against low-income countries supports concern for the lack of adequate systematic infrastructures and funding for surveillance and disease control in low-income countries, particularly regarding neglected zoonotic diseases, poverty alleviation, and emerging infectious diseases [10,27,28,29].

### 3.2. Description of Results by Type of Infectious Agent

**Figure 3:**
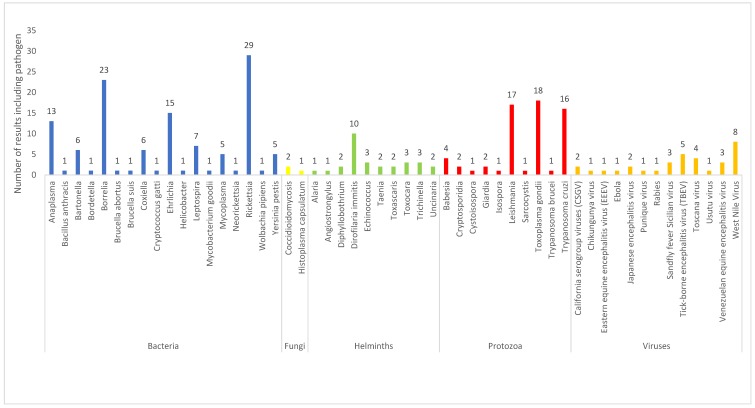
Pathogens identified from the results of the current review (*n* = 53).

Within the 142 results, 53 pathogens were described related to current or potential use of dogs as sentinel animals. Figure 3 shows distribution of pathogens identified, organized by infectious-agent type. For the purposes of this review, results have been summarized and synthesized for key pathogens. Key pathogens were selected as those with a high number of results in order to provide a comprehensive review as to how dogs may be utilized as sentinels, and are limited in number due to the scope of the paper.

#### 3.2.1. Viruses

Of all the pathogens, thirteen (25%) were viral. Of these, eight were mosquito-borne (California serogroup viruses (CSGV), Chikungunya virus, equine encephalitis virus (EEV), Japanese encephalitis virus (JEV), Toscana virus, Usutu virus, Venezuelan equine encephalomyelitis (VEE), and West Nile virus (WNV)), two were sandfly-borne (Punique virus and sandfly fever Sicilian virus (SFSV)), and one was tick-borne (tick-borne encephalitis virus (TBEV)). Two were not vector-borne (Ebola and rabies).

The West Nile virus was the most described viral pathogen, with eight references whose publication dates were in the range of 2001–2017. Countries of data collection were Canada (1), China (1), USA (3), Morocco (1), and Senegal (1) as individual studies, and one study compared data from France, Chad, Djibouti, Senegal, Côte d’Ivoire, Republic of the Congo, and Gabon [30]. Results from the USA and other countries are summarized below.

Seroprevalence was evaluated in healthy dogs, cats, and horses in New York City after the 1999 WNV outbreak in humans [31]. WNV antibodies were readily detected in dogs, with approximately 10% of dogs infected within certain boroughs, and an age-stratified analysis finding no evidence of long-term infection, thereby supporting their hypothesis that WNV was introduced in 1999. Although stray dogs had a higher seroprevalence compared to pets, this difference was not statistically significant. Similarly, a cross-sectional analysis during an epidemic in Slidell, Louisiana, United States screened 442 dogs for seropositivity to WNV, and found that 26% of dogs had positive results [32]. Outdoor-only family dogs had almost 19 times as great odds for seropositivity compared to indoor-only family dogs, and the odds for stray dogs were twice those of family dogs. It was also noted that family dogs that did not receive medication for heartworm were 2.5 times more likely to have a positive result compared to those who did. Results of weekly testing of juvenile stray dogs throughout WNV transmission season in Houston, Texas, detected positive dogs six weeks before the first human case in Houston, and the highest number of human cases were reported in two peaks, the third week of August and second week of September, both peaks coinciding with the highest-point prevalence in dogs [17].

Seroprevalence to WNV has been compared between military dogs and horses across ten sites in Morocco, finding 60% seroprevalence rates in horses and 62% in dogs. These findings suggest that dogs offer an alternative sentinel species to horses, particularly in areas where equine vaccination is used, thereby precluding passive surveillance of equine WNV cases [33]. A 2017 study conducted in southern Quebec, Canada was performed to assess public-health risk by estimating regional prevalence to WNV and other arboviruses in humans, horses, and dogs [34]. Results demonstrated sustained arboviral activity, and the seroprevalences of juvenile dogs indicated that virus transmission occurred in 2013 for WNV and California serotype-group viruses (CSGV). WNV seroprevalence of dogs has also been evaluated in Shanghai, results suggesting a higher seroprevalence in outdoor and rural pets compared with indoor and urban pets [35].

#### 3.2.2. Bacteria

Of all the pathogens, eighteen (34%) were bacterial. Of these, one was flea-borne (*Yersinia pestis*), four were tick-borne (*Anaplasma*, *Borrelia*, *Ehrlichia*, and *Rickettsia*), one was rarely tick-borne (*Coxiella*), one was transmitted by trematodes (*Neorickettsia*), three were transmitted by various arthropods (*Mycoplasma*, *Bartonella*, and *Wolbachia pepiens*), and eight were not vector-borne (*Bacillus anthracis*, *Bordetella*, *Brucella abortus*, *Brucella suis*, *Cryptococcus gatti*, *Helicobacter*, *Leptospira*, and *Mycobacterium goodii*).

Rickettsial disease was the most described bacterial pathogen, with 29 results, publication dates of which ranged from 1982 to 2017. Data were collected from Albania (1), Australia (1), Bolivia (1), Brazil (11), Cape Verde (1), Colombia (3), Costa Rica (1), Hungary (1), Israel (1), Spain (2), USA (5), and Zimbabwe (1). A serosurvey in dogs from North Carolina concluded that *R. rhiphicephali* was prevalent in the eastern coastal region, *R. montana* throughout the state, and *R. rickettsii* in the central Piedmont region but less so in the western mountains. Seroprevalence results for *R. rickettsii* approximated that of previously documented human exposure, and the authors concluded that dogs may be appropriate sentinels for determining the geographic prevalence of SFG rickettsia [36]. In 2001, a higher seroprevalence to *R. akari* in older New York dogs suggested that conditions in New York might have been favorable to the maintenance of *R. akari* ten or more years ago [37]. A case report of presumed fatal Rocky Mountain spotted fever (RMSF) in two dogs and their owner in Mississippi described how two dogs from the same household died from a suspected tick-borne disease eight days apart, followed by their owner two weeks later, necropsy samples from whom were positive for the spotted fever group *rickettsia* spp. [38]. An exploratory serosurvey to detect antibodies against SFG rickettsia in dogs sampled outside a previously documented RMSF outbreak area of Arizona concluded that the risk for human RMSF infection extended beyond previously known regions [39]. 

Studies in São Paulo, Brazil have suggested the use of dogs as sentinels by demonstrating higher seroprevalence of dogs in endemic regions as compared to nonendemic regions [40], and performing serosurveys of humans and dogs indicating the shared exposure to *R. rickettsii* by the *Amblyomma aureolatum* tick [41]. Data from the low endemic regions of Pingo D’Ahua and Santa Cruz do Escalvado support the importance of dogs as sentinels of rickettsial circulation in urban areas [42], and results from an endemic region of Rio de Janeiro State demonstrated a 2.8-fold higher probability of dogs exposed to forested areas producing rickettsial-group spotted-fever antibodies [43]. A serosurvey of dogs and cats in Caratinga was performed ten years after a previous serosurvey had been conducted in order to evaluate the success of vector control measures adopted by public health services, concluding that the vector control had been successful [44]. Occurrences of Brazilian spotted fever in the northeast of Paraná State prompted a risk assessment utilizing serosurveys of dogs, leading to the construction of risk-probability maps using seropositivity rates and known environmental aspects of the municipalities [45]. A seroprevalence study conducted in San José, Costa Rica demonstrated that dogs from areas associated with human cases of SFG rickettsia had greater odds of being seropositive than that of shelter dogs [46]. Of further note was one confirmed human case that was linked directly to a dog with a high-end titer seropositive dog, indicating shared exposure to the same vector. Another study from Zimbabwe determining prevalence *of R. conorii* found that there was a positive correlation between dog and human seroprevalence in some instances, but not all [47], concluding that, while dogs appear to be a sensitive indicator for the presence of *Rickettsia* within a region, seroprevalence of dogs should not be used to predict disease in humans.

#### 3.2.3. Protozoa

Of all the pathogens, ten (19%) were protozoal. Of these, one was sandfly-borne (*Leishmania*), one was tick-borne (*Babesia*), one was transmitted by the triatomine bug (*Trypanosoma cruzi*), and one was transmitted by the tsetse fly (*Trypanosoma brucei*). Six were not vector-borne (*Cryptosporidia*, *Cystoisospora*, *Giardia*, *Isospora*, *Sarcocystis*, and *Toxoplasma gondii*).

*Toxoplasma gondii* was the most described protozoal pathogen, with 17 papers published between 2004 and 2017. Data were collected from the following countries: Albania (1), Brazil (7), Canada (2), China (1), Egypt (1), Grenada (1), Mexico (2), Spain (1), and Uganda (1). Both papers from Canada were studying Indigenous communities, which are considered to have increased exposure to, and risk from, zoonotic parasites. High seroprevalence was found in dogs from Fort Chipewyan, Alberta, and Fort Resolution, Northwest Territories, indicating that *T. gondii* is common in the study areas, although sources of exposure were not identified [48]. Serosurveys of humans and dogs from two Saulteaux communities in southeastern Saskatchewan were conducted, which found 1.8% of humans and 21% of dogs had been exposed, supporting the use of dogs as sentinels for public health. Results identified age of the person, feeding raw meat to the dogs, and a history of not deworming pets to be exposure-risk factors [21]. Human and canine seroprevalence rates in Londrina, Brazil were compared [49]. Results concluded that, in the situation of urban toxoplasmosis, there was significantly higher seroprevalence in humans (42%) than their owned dogs (16%), and that seroprevalence of dogs was directly linked to increased numbers of dogs and dirty backyards. This suggests that, while dogs did not prove to be a good indicator of foodborne disease in people, they may be a reliable indicator of environmental infection. Other studies have also concluded that dogs may be used as environmental sentinels [50,51,52]. Results from a study in Ubatuba, São Paulo State [53] utilized dogs as sentinels to demonstrate that toxoplasmosis was linked to sanitary problems (largely cooking of food), rather than the presence of rodents or untreated water.

#### 3.2.4. Fungi

Of all the pathogens, only two (4%) were fungal; *Coccidioides* and *Histoplasma capsulatum*. Data were collected from the United States (2) and Brazil (1), and publication dates ranged from 2011 to 2017. A serosurvey of dogs from private veterinary clinics and a research control center in northeastern Brazil was performed, in which histoplasma antibodies were detected in 1.78% of serum samples, three of which were also positive for *Leishmania* spp. [54]. The remaining two studies both evaluated rates of coccidioidomycosis infection in American dogs to indicate risk for human infection [55,56]. Results demonstrated that areas with a high rate of coccidioidomycosis in dogs in Texas overlapped with those formerly identified as potential risk areas based on human surveys, and that there was significant correlation between reported human rates of infection and the generated risk map of canine coccidioidomycosis in California, thus providing evidence that dogs may be utilized as sentinels to describe the risk of coccidioidomycosis in humans.

#### 3.2.5. Helminths

Of all the pathogens, ten (19%) were helminths. Of these, one was mosquito-borne (*Dirofilaria immitis*), and nine were not vector-borne (*Alaria*, *Angiostrongylus*, *Diphyllobothrium*, *Echinococcus*, *Taenia*, *Toxascaris*, *Toxocara*, *Trichinella*, *Uncinaria*). Description of these pathogens has been limited to *D. immitis* alone, as the remaining pathogens were included in three or fewer studies. *D. immitis* was the most described helminth, with ten papers including it in the data, and publication dates ranging from 2007 to 2017. Data were collected from Albania (1), Australia (1), Brazil (2), Canada (2), Ecuador (1), Portugal (1), and the United States (2). All results were seroprevalence studies in dogs alone. The two Canadian studies did not find evidence of *Dirofilaria* in dogs from coastal British Columbia or southeastern Saskatchewan [21,57]. The two U.S. studies found 28% seroprevalence in rural dogs in northern California [58], and 3.9% across the southeast United States [59]. No positive samples were found from regions in Australia and Brazil [60,61,62]. Seroprevalences of 11.2% and 34% were found across Albania and the Galapagos, respectively [63,64]. Results from Portugal indicate that seropositivity to *D. immitis* is a risk factor for clinical signs consistent with canine vector-borne disease (including borreliosis, ehrlichiosis, anaplasmosis and leishmaniosis), with an odds ratio of 2:4 [65].

## 4. Review and Discussion of Dog-Sentinel Surveillance in Canada

### 4.1. Publications and First Nations (Indigenous) Communities in Canada

Publications with data collection from Canada are summarized in Table 2, overall accounting for six out of 142 publications (4% of the results). Of these, four of the papers focused on dogs from remote First Nations communities in Canada, which represent a distinct population of Canada and deserve special mention. While it is beyond the scope of this discussion to describe the history and current socioeconomic status of First Nations groups, it is recognized that veterinary services are often absent or restricted, and preventative healthcare very limited [48].

Typically, common practices in Canada such as vaccination, parasite prevention, and sterilization are often not accepted, and cultural barriers to accessing veterinary services, cost, and distance are also commonly cited problems [21]. Dogs in these remote communities are often free-roaming, with access to garbage, human food, and carcasses of fish and wildlife, and are therefore highly exposed to a variety of pathogens, making them ideal candidates for sentinel surveillance. However, they may also act as a source of infection (e.g., ingestion of *Toxocara* spp. eggs from fecal contamination), the recipient of human infection (e.g., *Giardia* spp. from human sewage), or as amplifiers (e.g., *Diphyllobothrium* spp. cestodes from raw or undercooked fish) [66].

Differences in husbandry and feeding preferences between First Nations groups are reflected in results from some of the studies. Examples include coastal dogs that were found to have lower prevalence of hookworm than northern sled dogs, most likely as they are usually not tied up, allowing feces to be deposited over a wider area [57], and lower levels of *Trichinella* in dogs belonging to Saulteaux communities, which typically cook their game and avoid bear meat, unlike others [21]. Therefore, it is important to consider the practices of each community individually, and not to extrapolate results from one to another.

### 4.2. Viruses

#### 4.2.1. Arboviruses

Arthropod viruses comprise 11 of the 13 viral results, some of which are present, or have potential to emerge in Canada. Mosquito-transmitted arboviruses that have been isolated in Canada include EEV, Western equine encephalitis (WEE), WNV, St. Louis encephalitis, California encephalitis, California serogroup viruses (such as the Jamestown Canyon virus and snowshoe hare virus), and Cache Valley virus. Viruses of concern to travelling Canadians include Zika, Chikungunya virus, dengue, yellow fever, Murray Valley, and Japanese encephalitis [68]. Vector-borne zoonotic viral pathogens considered to be of high risk of emergence in Canada include the Powassan virus, WNV, EEV, WEE, CSGV, and the Cache Valley virus [69].

Jamestown Canyon virus and snowshoe hare virus have been isolated across Canada [70], with 24 human cases confirmed in 2016 [71], and 20 types of mosquito able to transmit the viruses have been found across Canada, as far north as the Yukon and Northwest Territories [68]. Clinical signs are similar to those of WNV and contribute to a significant burden of disease in Canada. It has been suggested that CSGV may be under-recognized in Canada, and noted that arboviral encephalitis usually indicates the ‘tip of the iceberg’ with regards to the number of arboviral cases [72]. This is re-enforced by findings from the present review, which have suggested that physicians should consider CSGV as well as WNV in differential diagnosis of encephalitis based on seroprevalences in dogs (and horses) in Quebec, Canada [34]. Dogs have not been found to be efficient amplifier hosts of the Jamestown Canyon virus, which is another attribute for their use as sentinels [73].

The Chikungunya virus is transmitted by mosquitoes and is of concern for travelers coming back to Canada from various parts of the world, including the Caribbean. There is potential that the vector *Aedes albopictus*, as well as the virus, might become established given projected climate changes. The probability of autochthonous transmission in Canada considering recent and projected climate change has been assessed, and although the risk was found to be very low, it was noted that small areas of southern coastal British Columbia could become suitable in time, with up to one to two months of potential transmission per year [74]. Results suggest that antibodies in both dogs and humans develop at a similar rate [75]; therefore, these at-risk areas might be an ideal location to utilize local dog populations as sentinels for detecting the Chikungunya virus.

EEV is an immediately notifiable disease in Canada, so laboratories are required to report suspicion or diagnosis to the Canadian Food Inspection Agency (CFIA). In the summer of 2016, the first human case of EEV was reported in Ontario and made a full recovery. While human cases are rare, an estimated fatality rate of 35–75% makes it the deadliest mosquito-borne pathogen in North America [76]. EEV is thought to have become endemic in regions of Ontario and Quebec within the last ten years, and it has been suggested that the lack of confirmed human and equine cases may be due to the lack of diagnostic testing for the pathogen [34]. Researchers from Quebec found 0% of juvenile and 0% of adult dogs were seropositive to EEV, but they note that selecting dogs that have increased outdoor exposure might improve the sensitivity of a surveillance system [34]. Further study into the sampling of dogs for EEV serosurveillance is needed to support their use as sentinels.

JEV is only considered to be a risk for Canadians who are travelling to at-risk regions [77]. Likewise, VEE has never been reported in Canada, and is thought to be limited to South and Central American regions [78]. Research suggests that VEE is maintained in limited geographic regions, with only occasional spread to neighboring regions, likely due to limited mobility of mosquito vectors and rodent hosts [79], and therefore presents a low risk to Canada. Health Canada states there is no risk of contracting tick-borne encephalitis virus in Canada [80], and although there are reported cases of travel-related infection, no papers were found to suggest there is concern for emergence of this pathogen in Canada.

#### 4.2.2. West Nile Virus

The most widely studied arbovirus is WNV. Dogs may provide an ideal sentinel for WNV and potential human exposure, as they have similar vector-feeding patterns, they are susceptible to the infection but resistant to disease, develop antibodies that are easily measured, and do not develop viremia that is sufficient to infect mosquitoes or other species [32]. Although clinical cases have been reported in dogs, their short-term and low-level viremia means they are very unlikely to play an epidemiological role in the virus cycle [33].

It has been concluded that the presence of seropositive dogs does not necessarily suggest an increased risk for human infection, and dogs might even provide zooprophylaxis by diverting the bites of potentially infectious mosquitoes away from people [31]. The data from the former reference have previously been compared to results of a human serosurvey carried out in Queens, New York [81], which revealed that, at the same time and within the same area of Queens, 3% of humans surveyed were seropositive, whilst 11% of dogs were seropositive, highlighting the utility of dogs as sentinels for exposure to humans. It has been suggested that public-health departments could test a sample of captured stray dogs at animal control facilities weekly, so that results can be used to provide a warning of mammalian WNV transmission in the region to possibly aid prediction of human cases, and to track mosquito-population control measures [32].

Dogs might be a better choice of sentinel for WNV activity than birds, as the risk to humans increases when particular species of mosquitoes that act as ‘bridge vectors’ become abundant [31]. Therefore, using a mammalian sentinel may be more appropriate when estimating risk of human infection. While the currently used observation of bird mortality is effective in determining the presence of WNV, their high mobility and wide home ranges means their place of death does not necessarily correlate with site of infection. Furthermore, birds may develop resistance to disease leading to fewer deaths, rendering them less useful as a long-term surveillance tool [17].

WNV is currently endemic in several regions in southern Canada, and the government undertakes human, mosquito, bird, and horse surveillance, with 104 human cases confirmed in 2016 [82]. Results from this review suggest that dog-sentinel surveillance would be a useful addition to this strategy, not only to detect new areas of geographic expansion, but also to predict peaks in human-infection cases. The ideal dogs to monitor would be those spending more time outdoors, and who are not given prophylactic parasite control. The risk period for WNV infection in humans begins in mid-April and ends at the first hard frost in September/October, with the highest risk between mid-July and September [82]. Given the potential for dog antibodies to be detected up to six weeks before the first human case [17], sampling dogs from early March would be prudent. Currently, many dogs across Canada are tested annually for four vector-borne parasites (*Borrelia*, *D. immitis*, *Ehrlichia* spp., and *Anaplasma* spp.) between March and June (before starting mosquito prophylaxis), so this presents an ideal opportunity to add on testing for WNV in suitable dogs within or near endemic regions.

### 4.3. Bacteria

#### 4.3.1. Rocky Mountain Spotted Fever

Rocky Mountain spotted fever is present in western Canada, and a 2.5% seroprevalence has been documented in rural dogs of southeastern Alberta and southwestern Saskatchewan [83]. The disease has not been notifiable in Canada since 1978 [84], which has limited epidemiological understanding and potentially might limit resources and funding for future study. As described previously, there are many studies to demonstrate the use of canine serosurveys to indicate pathogen presence, distribution, and risk of human infection within one or more regions. However, it has also been highlighted through case reports of individual dogs and people that there is a need for increased communications between veterinarians and physicians on an individual case level to prevent disease or provide timely warning [38,85]. Serologic sampling of dogs may alert their handlers to the presence of the pathogen in their environment, and offer useful information for physicians of dog owners who present with unexplained febrile illness [85]. Dogs and their owners may share common exposures to tick-infested areas, dogs may transport ticks to closer proximity of their owners, and these ticks may establish themselves near the home, or even cause infection by direct handling of the tick by the owner. Stray dogs are more likely to have antibodies to SFG rickettsia than relinquished dogs, making them an appropriate group to sample [39].

#### 4.3.2. *Anaplasma*

Two out of 13 publications identified in this review were from Canada. Both sampled dogs from remote communities (coastal British Columbia and southeastern Saskatchewan) and neither found evidence of *Anaplasma* [21,57]. However, human granulocytic anaplasmosis may be of growing concern due to the establishment and expansion of *Ixodes scapularis* [69], and this pathogen has been isolated in three dogs from Saskatchewan with no history of travel [86]; therefore, further research into dog-sentinel surveillance is warranted in regions where *I. scapularis* is present. Results from multiple countries, including Albania, Australia, Ecuador, and the United States, have demonstrated the successful use of dogs as sentinels to indicate *Anaplasma* spp. [58,63,64,87].

#### 4.3.3. *Borrelia*

Lyme borreliosis is considered to be an emerging pathogen due to the northward expansion of the tick vector *I. scapularis* [69]. The speed of expansion of vector species has been faster than predicted [88], and invasion of *B. burgdorferi* in the eastern regions of this range usually occurs in the following three to five years [89]. Infected tick populations have now been documented in southern regions of Manitoba, Quebec, and Ontario, and in specific regions of New Brunswick and Nova Scotia [69], as well as British Columbia, where blacklegged ticks were found before other parts of Canada [90]. It is predicted that climate change will speed the expansion of Lyme borreliosis, increase the intensity of transmission, and could potentially alter the speed of bird migration and therefore distances of tick dispersion [91].

There is significant literature to support the use of dogs as sentinels for Lyme borreliosis, and such surveillance might also indicate the potential risk for other pathogens sharing the same vectors; for example, those causing granulocytic anaplasmosis, human babesiosis, Powassan encephalitis, and *Borrelia miyamotoi*, which are also transmitted by *Ixodes* ticks [69]. However, certain caveats must be considered when planning such surveillance systems. Results from the present review have highlighted that the movement and travel of dogs may affect results [92], and this may not always be easy to account for depending on the sampling of dogs. In addition, tick chemoprophylaxis, which is commonly used by dog owners, particularly in high-risk regions, reduces serosurvey sensitivity [93]. Current surveillance for Lyme disease in Canada incorporates reporting of human cases, active environmental tick surveillance, and passive surveillance of ticks voluntarily submitted by doctors and veterinarians [94]. Despite the large number of dogs that are routinely tested each year for *B. burgdorferi*, veterinary laboratory data are not included in current surveillance mechanisms. The standard diagnostic testing of *B. burgdorferi* in dogs typically involves initial screening by measurement of antibodies, followed by antigen detection to confirm active infection.

#### 4.3.4. *Ehrlichia*

Ehrlichiosis is considered to be a high-risk emerging zoonosis in Canada [69], with human cases reported widely across regions of the United States [95], and a new pathogenic species was detected in the bordering states of Minnesota and Wisconsin [96]. Two of the results from the present review utilized dog serosurveys in Canada (southeastern Saskatchewan and coastal British Columbia) in surveys for *Ehrlichia*, both of which did not find a positive result [21,57]. However, it should be noted that both these studies utilized small sample sizes. Focusing sentinel surveillance in high-risk areas for emerging disease, such as southern Manitoba and southwestern Ontario, is likely to be more productive. Results from the present review, which found infection in surveillance dogs, include symptomatic dogs from a veterinary referral hospital in Brazil [61], urban domestic dogs in Brazil [97], owned domestic dogs in the Cape Verde archipelago [98], samples submitted to diagnostic laboratories across the United States [59], and asymptomatic dogs presented to veterinary clinics in the United States [99]. Therefore, utilizing owned domestic dogs, and testing convenient samples from laboratories or referral hospitals is likely to be an efficient method of undertaking sentinel surveillance.

### 4.4. Protozoa

#### 4.4.1. *Toxoplasma gondii*

Globally, *T. gondii* is one of the most common foodborne parasites, causing an estimated 10.3 million cases and 825,000 disability-adjusted life years (DALYs) in 2010 [100]. Across different regions of Canada, serosurveys have revealed seroprevalences ranging from 6.2% to 61.2% [101]. It is not reportable or immediately notifiable in Canada, although it is one of several pathogens for which reports must be annually submitted to the World Health Organization (WHO), and veterinary laboratories are obligated to comment on Canada’s report to the World Organization for Animal Health (OIE) [102]. There has been a focus on Indigenous communities, and in particular Arctic communities, where, even in the absence of feline hosts, migratory wildlife and spring melting water flow contribute to parasite transmission between ecosystems [103]. The two Canadian results from this review (described previously) were from Indigenous communities and supported the use of dogs as sentinels, but it is important to emphasize that they serve as environmental sentinels.

#### 4.4.2. *Babesia*

In North America, human babesiosis is largely caused by *B. duncani* and *B. microti*, and is considered an emerging disease in Canada, with recent first-time reports of locally acquired *B. microti* and *B. duncani* infection in Manitoba and Ontario, respectively [69,104,105]. There is a risk of infection via blood transfusions, and such infection has been detected in U.S. blood donors close to Canadian borders [106]. Results of the present review show dogs have been used as sentinels to detect *B. vogeli* in Australia and southern Brazil [87,97]. While there have been no reports (to the authors’ knowledge) of canine infection with *B. microti* or *B. duncani*, the surveillance of *Babesia* spp. is expected to provide information on expanding tick habitats, as well as potentially identifying new species.

### 4.5. Helminths

#### *Dirofilaria immitis* 

While neither of the papers from the present review documented infection in Canadian dogs, there is low prevalence of the disease in Canada, with most positive dogs found in southern Ontario, southern Manitoba, southern Quebec, and the southern Okanagan Valley [107]. In 2017, Idexx veterinary laboratories reported 285 positive cases in Ontario, 39 in Manitoba, 30 in Quebec, and much fewer cases in New Brunswick, Nova Scotia, and Prince Edward Island [108], which was an overall increase compared with 2016. *D. immitis* is pathogenic to humans via the bite of an infected mosquito. Clinical signs and radiographic thoracic lesions are not specific for the pathogen, which often leads to invasive diagnostic procedures to rule out other diagnoses such as cancer [109]. There were 81 cases reported in the United States between 1941 and 2005 [110]. The publication demonstrates how human cases were shown to reflect the prevalence of *Dirofilaria* in dogs in the United States, and also how the veterinary literature has informed current medical knowledge of human cases. Despite a current lack of reports of Canadian-acquired human infection, the known prevalence in dogs in southern Ontario and Quebec alongside the presence of human cases in the bordering states of Wisconsin, Michigan, and New York State could imply increased risk for human infection. Sentinel surveillance of canine populations in these regions may be able to predict and inform health authorities of increasing infection rates.

### 4.6. Fungi

While there were very few results evaluating dog-sentinel surveillance for fungal pathogens, the results indicated that such surveillance may be useful when evaluating risk of infection in humans from environmental pathogens such as histoplasmosis and coccidioidomycosis. Histoplasmosis was thought to be limited to central regions of Canada along the St Lawrence River; however, a cluster in Alberta, western Canada was reported in 2003 [111]. Occupations involving working with soil, and an immune-compromised status increase the risk of infection. Coccidioidomycosis (valley fever) is currently thought not to be present in Canada, and reported human or animal cases in Canada have been associated with travel (predominantly to the United States) [112]. Although it has been suggested that no Canadian region is ecologically suited to the establishment of this pathogen [112], expert opinion appears to be less certain about the influence of climate on such pathogens [113]; therefore, this risk should be monitored in the future.

## 5. Limitations of the Review

Outcome-reporting bias and publication bias are recognized potential flaws of systematic reviews and meta-analyses; positive outcomes are more likely to be reported, and studies with significant or interesting conclusions are more likely to be accepted for publication, both of which may bias the results of a literature review [114,115]. The results of this study yielded only four papers that did not support the use of dogs as sentinels, so it is likely this review has been subject to such bias. In addition, this review only included papers published in their entirety in English, which may have significantly limited the results. The Sub-Saharan Africa and Middle East/North Africa regions are under-represented, but it is not possible to determine if this is due to restricting language to English or a true disparity. Repeating the search process to include other languages would help clarify this. Funding for the publications was not examined due to the complexity and ambiguity of funding sources. Opportunities for funding of research projects are more limited in low-income countries and this will have contributed to the under-representation of publications from the regions above.

The study design and quality of each paper were not assessed for the purposes of inclusion or exclusion, as the objective of this review was to scope out all current knowledge and research on the subject rather than to make concrete observations or measurements. Publications were assessed at a qualitative level due to the scoping nature of the review, and quantitative measures such as disease prevalence were not specifically evaluated.

## 6. Implementing a Dog-Sentinel Surveillance System: General Principles and Limitations

Translating the theoretical idea of sentinel surveillance into a feasible and practical surveillance system requires examination of several factors. Firstly, the objective of the surveillance must be clear; for example, whether the objective is to measure frequency of disease or to provide a warning of disease emergence or expansion will determine which regions and dog populations will be most useful. The region(s) should be selected based on known or estimated prevalence of disease, or presence or risk of vector emergence, and sentinel units (e.g., veterinary clinics, shelters, and laboratories) selected to maximize the included population. Dog populations utilized would depend on the specific pathogen of concern and might include live sampling of dogs or the use of samples already taken for other diagnostic tests. Sampling strategy would be formed based on the objective of the study as well. For example, if the objective is to detect a new wave of viral transmission, then repeatedly testing naive juvenile dogs would provide an ideal sample, whereas, if measuring prevalence of a rare disease, dogs at high risk for exposure should be selected. The selected dog populations should be based on their availability for sampling, increased susceptibility to the pathogen in question, relationship to the pathogen and human population they are to represent, and the number of dogs available to sample. It is also important to note that, when samples represent a subset of clinically ill dogs, measured prevalence cannot be used to estimate regional prevalence.

Constraints such as time, cost, risks to research staff, and logistical feasibility should be considered. The ethical aspects of utilizing dogs as sentinels must also be thought through when designing an active surveillance system or conducting any type of research or testing [116]. While the nature of utilizing dogs as sentinels precludes replacement, it might be possible to reduce the sample size by selecting dogs with increased risk exposure and to refine procedures by minimizing the number of times a dog is sampled. Ideally, tests would be performed on samples that had already been taken for a different purpose, or added on to a sample that would be taken anyway. Utilizing samples from a veterinary laboratory or asking veterinary staff to add on a test to future samples would be a way of achieving this. Many dog shelters would routinely test newly admitted dogs for a range of parasitic diseases, which can provide an opportunity to add on other tests.

There are limitations to the use of dog-sentinel surveillance that need to be considered when designing surveillance schemes and analyzing data outputs. Many variables cannot always be accounted for, such as travel history, previous medications (including prophylactic treatment), animal movements and owner compliance. Travel of dogs across regions and borders in particular affects data interpretation; ideally, anomalous results would be traceable to the animal so that a history could be obtained. The nature of the diagnostic test used would also aid interpretation of a positive result, for example, whether the test suggests active infection rather than previous exposure. Furthermore, animal-sentinel surveillance is an indirect method of measuring risk to human disease, and it is difficult to translate data obtained from such surveillance into a measured risk to humans. Therefore, it would be prudent to focus on the trends, patterns, and emergence of pathogens rather than to try and quantify risk. While not a limitation of dogs as sentinels, there is also the issue of funding sources, as it is not immediately obvious whether a sentinel-surveillance study of companion animals would fall under the mandate of the CFIA, which is typically concerned with animal health and food safety, or the Public Health Agency of Canada (PHAC), which includes disease prevention and response to public-health threats in its activities. This might be decided on a provincial basis and thus could vary across Canada.

## 7. Recommendations

The objectives of this review were to identify the available literature supporting the use of dogs as sentinels so that further, more targeted research could be planned to assist with its implementation. Anticipated actions resulting from such sentinel surveillance include warnings of pathogen activity to physicians in order to improve detection of human cases and consideration of other differential diagnoses, and to epidemiologists in order to detect ongoing and new pathogen or vector activity and predict peaks in transmission and human cases.

The review has described the major pathogens that are present, emerging, or at risk of emergence in Canada, with potential for dog-sentinel surveillance, based on existing research. Specific pathogens that present a risk to Canadian populations have been summarized in Table 3, and the authors suggest that these are evaluated further with regards to dog-sentinel surveillance in the appropriate geographic regions. This evaluation would need to consider the availability of an adequate sample population, efficient diagnostic testing tools, resources, and personnel to collect and process the data to provide a meaningful assessment of pathogen presence and risks to human health, and the communication channels to share these findings and potential warnings to the appropriate recipients.

More generally, this review has also reinforced the value and need for greater communication and collaboration between the human and veterinary medical sectors in a One Health manner, whether that be by communicating epidemiological data within a region or, even better, to have standardized data formatting so human and animal data could be entered into a shared database, as well as considering the health of owned dogs on an individual-case basis. Legally mandated reporting of certain diseases that are already monitored in dogs, such as Lyme borreliosis, would provide a good means of introducing this initiative.

## 8. Conclusions

In this study, a systematic-review method was used to identify publications related to the global use of dogs as sentinels for human infectious disease. A total of 142 results were included, encompassing 53 pathogens. Review of this literature revealed some biases: 60% of all data originated from the Latin American, North American, and Caribbean regions, and 89% of data originated from high-income and upper-middle-income countries. The number of studies published per year has increased over time, suggesting a growing interest in the subject. Bacterial, protozoal, and viral infectious agents were the most researched infectious-agent types, accounting for 34%, 29%, and 25% of all pathogens, respectively.

Dogs in Canada are currently underutilized as sentinels for human infectious disease, yet carry enormous potential as a sensitive and cost-effective method of disease surveillance. The majority of research conducted in Canada has been within First Nations (Indigenous) communities. Dogs may serve as sentinels for environmental pathogens, such as toxoplasmosis and histoplasmosis, for pathogen or vector expansion of endemic pathogens such as *B. burgdorferi*, or emerging pathogens such as the Chikungunya virus and California serogroup viruses. Further study and application of dog-sentinel surveillance is recommended for California serogroup viruses, Chikungunya virus, West Nile virus, Lyme borreliosis, *Rickettsia* spp. *Ehrlichia* spp. and *Dirofilaria immitis*.

Whilst there has been limited research undertaken to evaluate the potential of dogs as sentinels, more targeted study of the suggested pathogens and regions is required so that we may move past serosurveillance studies and develop active surveillance systems to inform and exact action across veterinary and human health within a One Health framework. From a global perspective, the significant disparity between high- and low-income economies has again been demonstrated, reinforcing the need for improved funding and infrastructure development for disease surveillance in low-income economies.

## Figures and Tables

**Table 1:** Search terms used in the search process.

Term	Search Terms and Synonyms Used
dog	“dog*” OR “canine” OR “canis”
sentinel	“sentinel*” OR “indicator*”
disease	“public health” OR “infectious disease” OR “zoono*” OR “epidemiolog*”

**Table 2:** Results with data originating from Canada.

Pathogen(s) Studied	Location	Populations	Conclusion	Reference
*B. burgdorferi*	British Columbia; regions with previous tick infestation	Domestic owned dogs: healthy, post tick-bite or symptomatic for tick-borne disease	Value of randomly sampled, asymptomatic dogs as sentinels is limited.	[67]
Multiple; viral, bacterial, helminths, protozoa	British Columbia; remote coastal regions	Dogs owned by First Nations communities	Provides baseline results for future monitoring of infectious agents that could affect dogs, wildlife, and humans.	[57]
Arboviruses: West Nile virus (WNV), equine encephalitis virus (EEV), California serogroup viruses (CSGV)	Southern Quebec	Domestic owned dogs	Dogs provide sensitive indication of past or ongoing WNV or CSGV activity, and can indicate when transmission occurred.	[34]
Multiple; protozoa, helminths	Alberta and Northwest Territories	Dogs owned by First Nations communities	Dogs may serve as sources and sentinels for parasites in people and wildlife, and as parasite bridges between wildlife and humans.	[48]
Multiple; protozoa, helminths	Alberta and Saskatchewan	Dogs owned by First Nations communities	Companion-animal surveillance of parasites is a potential tool for detection of zoonotic risks for people, and could be used to evaluate efficacy of animal and public health interventions.	[66]
Multiple; bacteria, helminths, protozoa	Southeastern Saskatchewan	Dogs owned by First Nations communities	Emphasized the use of dogs as sentinels for emerging pathogens and the need for targeted surveillance and intervention programs within cultural communities.	[21]

**Table 3:** Pathogens presenting a risk to Canadians, which could be monitored using dog-sentinel surveillance.

Pathogen	Status in Canada	Suggested Region of Surveillance	Risk(s) Being Assessed	Suggested Dog Samples or Populations for Sentinel Surveillance
California serogroup viruses	Considered to have high risk of emergence. Low prevalence currently, with vectors present. Not notifiable. 24 human cases confirmed in Canada in 2016.	Canada-wide	Emergence of pathogen.	Dogs of all ages for estimating period of former viral transmission. Juvenile dogs for detecting new periods of transmission.
Chikungunya virus	Low risk of emergence in one region, where climate change could enable establishment of the vector and virus for one to two months per year. Not notifiable.	Southern coastal British Columbia	Emergence of pathogen.	Outdoor dogs, preferably not receiving mosquito prophylaxis.
West Nile virus	Endemic in several regions of Canada with 104 human cases reported in 2016. Immediately notifiable; all laboratories must notify the Canadian Food Inspection Agency (CFIA) when suspecting or diagnosing disease.	Southern Canada	Expansion, risk of infection, and predicting rise in human cases.	Outdoor, rural, or urban dogs, preferably not receiving mosquito prophylaxis. Juvenile dogs for detecting and predicting new viral transmission. Serum taken from annual parasite checks could be analyzed for WNV.
*Rickettsia* spp.	Present in western Canada. Not notifiable since 1978.	Western Canada	Geographic prevalence and expansion, risk of human infection, and individual risk based on health of in-contact dogs.	Rural dogs, ideally not taking tick prophylaxis, and on an individual-case level, dogs in-contact with clinically ill humans.
Lyme borreliosis	Endemic in multiple regions across Canada. Notifiable disease in people since 2009.	Canada-wide	Expansion of pathogen, risk of infection, presence of vector that might indicate other diseases.	Passive surveillance of submitted ticks could be supplemented by mandatory reporting of positive Lyme cases by laboratories to human health sector.
*Ehrlichia* spp.	Considered to be high risk for emergence, with new pathogenic species detected in Minnesota and Wisconsin. Not notifiable.	Southern Manitoba and southern Ontario	Emergence of pathogen.	Dogs not receiving tick prophylaxis (free-roaming, outdoors, stray, or relinquished dogs) and those with clinical signs of a history of tick bites.
*Dirofilaria immitis*	Low prevalence in dogs. Not notifiable. No reports of Canadian human cases, but disease documented in Wisconsin, Michigan, and New York State.	Southern Ontario and southern Quebec	Expansion of pathogen, increased risk of human infection.	Laboratory results from annual parasite screening could be shared with the human health sector.

## Supplementary Materials

The following are available online at http://www.mdpi.com/2306-7381/5/4/83/s1, File S1: Full list of results of the literature search, Table S1: Example of data-capture form.
