# Supplementary material for: Dogs (Canis familiaris) as Sentinels for Human Infectious Disease and Application to Canadian Populations: A Systematic Review
Source: Vet Sci. 2018 Sep 21;5(4):83. doi: 10.3390/vetsci5040083 (PMC6313866; doi:10.3390/vetsci5040083)
Supplement: Supplementary file 1 [file vetsci-05-00083-s001.zip › S1 Full list of results of the literature search.rtf]

1.	Allela, L.; Bourry, O.; Pouillot, R.; Delicat, A.; Yaba, P.; Kumulungui, B.; Rouquet, P.; Gonzalez, J.-P.; Leroy, E.M. Ebola virus antibody prevalence in dogs and human risk. Emerging Infectious Diseases 2005, 11, 385.
2.	Alvarado-Esquivel, C.; Romero-Salas, D.; Cruz-Romero, A.; Garcia-Vazquez, Z.; Peniche-Cardena, A.; Ibarra-Priego, N.; Ahuja-Aguirre, C.; Perez-de-Leon, A.A.; Dubey, J.P. High prevalence of toxoplasma gondii antibodies in dogs in veracruz, mexico. BMC Veterinary Research 2014, 10, 191.
3.	Alwassouf, S.; Maia, C.; Ayhan, N.; Coimbra, M.; Cristovao, J.M.; Richet, H.; Bichaud, L.; Campino, L.; Charrel, R.N. Neutralization-based seroprevalence of toscana virus and sandfly fever sicilian virus in dogs and cats from portugal. Journal of General Virology 2016, 97, 2816-2823.
4.	Amal, S.M.S.; Asmaa, A.A.H.; Ismail, A.A.; Nasr, S.E.M.; Yasukazu, M.; Ueno, H.; Morita, C. Prevalence of coxiella burnetii infection among dogs and humans in upper egypt. Assiut Veterinary Medical Journal 2002, 47, 205-215.
5.	Anez, N.; Rojas, A.; Crisante, G.; Guevara, P.; Ramirez, J.L. Use of sentinel animals to demonstrate active leishmanial transmission in an area with low frequency of human lesions in western venezuela. Revista de Patologia Tropical 2003, 32, 63-72.
6.	Arce-Fonseca, M.; Carrillo-Sanchez, S.C.; Molina-Barrios, R.M.; Martinez-Cruz, M.; Cedillo-Cobian, J.R.; Henao-Diaz, Y.A.; Rodriguez-Morales, O. Seropositivity for trypanosoma cruzi in domestic dogs from sonora, mexico. Infectious Diseases of Poverty 2017, 6.
7.	Baek, B.K.; Lim, C.W.; Rahman, M.S.; Kim, C.H.; Oluoch, A.; Kakoma, I. Brucella abortus infection in indigenous korean dogs. Canadian Journal of Veterinary Research 2003, 67, 312.
8.	Banerjee, S.; Stephen, C.; Fernando, K.; Coffey, S.; Dong, M. Evaluation of dogs as sero-indicators of the geographic distribution of lyme borreliosis in british columbia. Canadian Veterinary Journal 1996, 37, 168-169.
9.	Baneth, G.; Nachum-Biala, Y.; Halperin, T.; Hershko, Y.; Kleinerman, G.; Anug, Y.; Abdeen, Z.; Lavy, E.; Aroch, I.; Straubinger, R.K. Borrelia persica infection in dogs and cats: Clinical manifestations, clinicopathological findings and genetic characterization. Parasites & Vectors 2016, 9, 244.
10.	Barbabosa-Pliego, A.; Gil, P.C.; Hernandez, D.O.; Aparicio-Burgos, J.E.; de Oca-Jimenez, R.M.; Martinez-Castaneda, J.S.; Ochoa-Garcia, L.; Guzman-Bracho, C.; Estrada-Franco, J.G.; Garg, N.J., et al. Prevalence of trypanosoma cruzi in dogs (canis familiaris) and triatomines during 2008 in a sanitary region of the state of mexico, mexico. Vector-Borne and Zoonotic Diseases 2011, 11, 151-156.
11.	Batista, F.G.; da Silva, D.M.; Green, K.T.; Tezza, L.B.D.; de Vasconcelos, S.P.; de Carvalho, S.G.S.; Silveira, I.; Moraes, J.; Labruna, M.B.; Fortes, F.S., et al. Serological survey of rickettsia sp in horses and dogs in an non-endemic area in brazil. Revista Brasileira De Parasitologia Veterinaria 2010, 19, 205-209.
12.	Benitez, A.D.; Martins, F.D.C.; Mareze, M.; Santos, N.J.R.; Ferreira, F.P.; Martins, C.M.; Garcia, J.L.; Mitsuka-Bregano, R.; Freire, R.L.; Biondo, A.W., et al. Spatial and simultaneous representative seroprevalence of anti-toxoplasma gondii antibodies in owners and their domiciled dogs in a major city of southern brazil. PLoS One 2017, 12, p.e.0180906.
13.	Bennett, M.D.; Abdad, M.Y.; Stenos, J. Serological evidence of rickettsia spp. In western australian dogs. The American Journal of Tropical Medicine and Hygiene 2017, 97, 407-412.
14.	Bowman, D.; Little, S.E.; Lorentzen, L.; Shields, J.; Sullivan, M.P.; Carlin, E.P. Prevalence and geographic distribution of dirofilaria immitis, borrelia burgdorferi, ehrlichia canis, and anaplasma phagocytophilum in dogs in the united states: Results of a national clinic-based serologic survey. Veterinary Parasitology 2009, 160, 138-148.
15.	Breitschwerdt, E.B.; Moncol, D.J.; Corbett, W.T.; MacCormack, J.N.; Burgdorfer, W.; Ford, R.B.; Levy, M.G. Antibodies to spotted fever-group rickettsiae in dogs in north carolina. American Journal of Veterinary Research 1987, 48, 1436-1440.
16.	Bryan, H.M.; Darimont, C.T.; Paquet, P.C.; Ellis, J.A.; Goji, N.; Gouix, M.; Smits, J.E. Exposure to infectious agents in dogs in remote coastal british columbia: Possible sentinels of diseases in wildlife and humans. Canadian Journal of Veterinary Research-Revue Canadienne De Recherche Veterinaire 2011, 75, 11-17.
17.	Cabezon, O.; Millan, J.; Gomis, M.; Dubey, J.P.; Ferroglio, E.; Almeria, S. Kennel dogs as sentinels of leishmania infantum, toxoplasma gondii, and neospora caninum in majorca island, spain. Parasitology Research 2010, 107, 1505-1508.
18.	Camer, G.A.; Park, H.; Roque, R.B.; Masangkay, J.S. Gastric helicobacter species in philippine dogs. Philippine Journal of Veterinary Medicine 2010, 47, 49-53.
19.	Cardinal, M.V.; Castañera, M.B.; Lauricella, M.A.; Cecere, M.C.; Ceballos, L.A.; Vazquez-Prokopec, G.M.; Kitron, U.; GÜRtler, R.E. A prospective study of the effects of sustained vector surveillance following community-wide insecticide application on trypanosoma cruzi infection of dogs and cats in rural northwestern argentina. The American Journal of Tropical Medicine and Hygiene 2006, 75, 753-761.
20.	Cardoso, L.; Mendao, C.; Carvalho, L.M.d. Prevalence of dirofilaria immitis, ehrlichia canis, borrelia burgdorferi sensu lato, anaplasma spp. And leishmania infantum in apparently healthy and cvbd-suspect dogs in portugal - a national serological study. Parasites and Vectors 2012, 5, 555.
21.	Castañera, M.B.; Lauricella, M.A.; Chuit, R.; Gürtler, R.E. Evaluation of dogs as sentinels of the transmission of trypanosoma cruzi in a rural area of north-western argentina. Annals of Tropical Medicine & Parasitology 1998, 92, 671-683.
22.	Castillo-Neyra, R.; Chu, L.C.; Quispe-Machaca, V.; Ancca-Juarez, J.; Chavez, F.S.M.; Mazuelos, M.B.; Naquira, C.; Bern, C.; Gilman, R.H.; Levy, M.Z. The potential of canine sentinels for reemerging trypanosoma cruzi transmission. Preventive Veterinary Medicine 2015, 120, 349-356.
23.	Coffey, L.L.; Crawford, C.; Dee, J.; Miller, R.; Freier, J.; Weaver, S.C. Serologic evidence of widespread everglades virus activity in dogs, florida. (special issue: Zoonotic diseases.). Emerging Infectious Diseases 2006, 12, 1873-1879.
24.	Comer, J.A.; Vargas, M.C.; Poshni, I.; Childs, J.E. Serologic evidence of rickettsia akari infection among dogs in a metropolitan city. Journal of the American Veterinary Medical Association 2001, 218, 1780-1786.
25.	Constantino, C.; Pasquali, A.K.S.; Caldart, E.T.; Ferreira, F.P.; Marana, E.R.M.; Freire, R.L.; Mitsuka-Bregano, R.; Hilst, C.L.S.; Vidotto, O.; Navarro, I.T. Seroepidemiology of leishmania spp. In dogs residing in telemaco borba, parana, brazil. Semina: Ciencias Agrarias 2014, 35, 3181-3190.
26.	Constantino, C.; Paula, E.F.E.d.; Brandao, A.P.D.; Ferreira, F.; Vieira, R.F.d.C.; Biondo, A.W. Survey of spatial distribution of vector-borne disease in neighborhood dogs in southern brazil. Open Veterinary Journal 2017, 7, 50-56.
27.	Constantino, C.; Pellizzaro, M.; Paula, E.F.E.d.; Vieira, T.S.W.J.; Brandao, A.P.D.; Ferreira, F.; Vieira, R.F.d.C.; Langoni, H.; Biondo, A.W. Serosurvey for leishmania spp., toxoplasma gondii, trypanosoma cruzi and neospora caninum in neighborhood dogs in curitiba-parana, brazil. Brazilian Journal of Veterinary Parasitology 2016, 25, 504-510.
28.	Cordeiro, R.A.; Coelho, C.G.V.; Brilhante, R.S.N.; Sidrim, J.J.C.; Castelo-Branco, D.; Moura, F.B.P.; Rocha, F.A.C.; Rocha, M.F.G. Serological evidence of histoplasma capsulatum infection among dogs with leishmaniasis in brazil. Acta Tropica 2011, 119, 203-205.
29.	Corredor, A.; Gallego, J.F.; Tesh, R.B.; Morales, A.; Ferro de Carrasquilla, C.; Young, D.G.; Kreutzer, R.D.; Boshell, J.; Palau, M.T.; Caceres, E., et al. Epidemiology of visceral leishmaniasis in colombia. American Journal of Tropical Medicine and Hygiene 1989, 40, 480-486.
30.	Costa, V.M.d.; Silva, R.d.C.; Branti, J.L.; Modolo, J.R.; Langoni, H. Prevalence and geographical distribution of toxoplasma gondii in dogs in the urban area of botucatu, sp, brazil. Brazilian Journal of Veterinary Research and Animal Science 2013, 50, 152-155.
31.	Cunha, N.C.d.; Lemos, E.R.S.d.; Rozental, T.; Teixeira, R.C.; Cordeiro, M.D.; Lisboa, R.S.; Favacho, A.R.; Barreira, J.D.; Rezende, J.d.; Fonseca, A.H.d. Rickettsiae of the spotted fever group in dogs, horses and ticks: An epidemiological study in an endemic region of the state of rio de janeiro, brazil. Revista Brasileira de Medicina Veterinaria 2014, 36, 294-300.
32.	da Costa, A.P.; Costa, F.B.; Soares, H.S.; Ramirez, D.G.; Araujo, A.D.; Ferreira, J.; Tonhosolo, R.; Dias, R.A.; Gennari, S.M.; Marcili, A. Environmental factors and ecosystems associated with canine visceral leishmaniasis in northeastern brazil. Vector-Borne and Zoonotic Diseases 2015, 15, 765-774.
33.	da Costa, L.; Caldart, E.T.; Ruffolo, B.B.; Toledo, R.D.; Dias, R.C.F.; Navarro, I.T.; Mitsuka-Bregan, R.; Vidotto, O.; Freire, R.L. Leishmaniasis in dogs from recycling centers and from a neighborhood with adjacent forest in an urban area of londrina, parana, brazil. Semina-Ciencias Agrarias 2016, 37, 1407-1414.
34.	da Silva, R.C.; de Lima, V.Y.; Tanaka, E.M.; da Silva, A.V.; de Souza, L.C.; Langoni, H. Risk factors and presence of antibodies to toxoplasma gondii in dogs from the coast of sao paulo state, brazil. Pesquisa Veterinaria Brasileira 2010, 30, 161-166.
35.	Dahmani, M.; Alwassouf, S.; Grech-Angelini, S.; Marie, J.L.; Davoust, B.; Charrel, R.N. Seroprevalence of toscana virus in dogs from corsica, france. Parasites and Vectors 2016, 9, 381.
36.	Davoust, B.; Leparc-Goffart, I.; Demoncheaux, J.P.; Tine, R.; Diarra, M.; Trombini, G.; Mediannikov, O.; Marie, J.L. Serologic surveillance for west nile virus in dogs, africa. Emerging Infectious Diseases 2014, 20, 1415-1417.
37.	Davoust, B.; Maquart, M.; Roqueplo, C.; Gravier, P.; Sambou, M.; Mediannikov, O.; Leparc-Goffart, I. Serological survey of west nile virus in domestic animals from northwest senegal. Vector-Borne and Zoonotic Diseases 2016, 16, 359-361.
38.	de Paiva Diniz, P.P.; Schwartz, D.S.; de Morais, H.S.; Breitschwerdt, E.B. Surveillance for zoonotic vector-borne infections using sick dogs from southeastern brazil. Vector-Borne and Zoonotic Diseases 2007, 7, 689-697.
39.	Dickerman, R.W.; Scherer, W.F.; Navarrom, E.; Ordonez, J.v. The involvement of dogs in endemic cycles of venezuelan encephalitis virus. American Journal of Epidemiology 1973, 98, 311-314.
40.	Diniz, P.; Morton, B.A.; Tngrian, M.; Kachani, M.; Barron, E.A.; Gavidia, C.M.; Gilman, R.H.; Angulo, N.P.; Brenner, E.C.; Lerner, R., et al. Infection of domestic dogs in peru by zoonotic bartonella species: A cross-sectional prevalence study of 219 asymptomatic dogs. Plos Neglected Tropical Diseases 2013, 7, p.e2393.
41.	Dubey, J.P.; Tiwari, K.; Chikweto, A.; DeAllie, C.; Sharma, R.; Thomas, D.; Choudhary, S.; Ferreira, L.R.; Oliveira, S.; Verma, S.K., et al. Isolation and rflp genotyping of toxoplasma gondii from the domestic dogs (canis familiaris) from grenada, west indies revealed high genetic variability. Veterinary Parasitology 2013, 197, 623-626.
42.	Duncan, A.W.; Correa, M.T.; Levine, J.F.; Breitschwerdt, E.B. The dog as a sentinel for human infection: Prevalence of borrelia burgdorferi c6 antibodies in dogs from southeastern and mid-atlantic states. Vector Borne and Zoonotic Diseases 2004, 4, 221-229.
43.	Durand, B.; Haskouri, H.; Lowenski, S.; Vachiery, N.; Beck, C.; Lecollinet, S. Seroprevalence of west nile and usutu viruses in military working horses and dogs, morocco, 2012: Dog as an alternative wnv sentinel species? Epidemiology and Infection 2016, 144, 1857-1864.
44.	El Behairy, A.M.; Choudhary, S.; Ferreira, L.R.; Kwok, O.C.H.; Hilali, M.; Su, C.; Dubey, J.P. Genetic characterization of viable toxoplasma gondii isolates from stray dogs from giza, egypt. Veterinary Parasitology 2013, 193, 25-29.
45.	Elchos, B.N.; Goddard, J. Implications of presumptive fatal rocky mountain spotted fever in two dogs and their owner. Journal of the American Veterinary Medical Association 2003, 223, 1450-1452, 1433.
46.	Eng, T.R.; Wilson, M.L.; Spielman, A.; Lastavica, C.C. Greater risk of borrelia burgdorferi infection in dogs than in people. Journal of Infectious Diseases 1988, 158, 1410-1411.
47.	Esamaeili, S.; Azadmanesh, K.; Naddaf, S.R.; Rajerison, M.; Carniel, E.; Mostafavi, E. Serologic survey of plague in animals, western iran. Emerging Infectious Diseases 2013, 19, 1549-1551.
48.	Foley, J.E.; Foley, P.; Madigan, J.E. Spatial distribution of seropositivity to the causative agent of granulocytic ehrlichiosis in dogs in california. American Journal of Veterinary Research 2001, 62, 1599-1605.
49.	Fortes, F.S.; Silveira, I.; Moraes, J.; Leite, R.V.; Bonacim, J.E.; Biondo, A.W.; Labruna, M.B.; Molento, M.B. Seroprevalence of rickettsia bellii and rickettsia felis in dogs, sao jose dos pinhais, state of parana, brazil. Revista Brasileira De Parasitologia Veterinaria 2010, 19, 222-227.
50.	Galvao, M.A.M.; Cardoso, L.D.; Mafra, C.L.; Calic, S.B.; Walker, D.H. Revisiting brazilian spotted fever focus of caratinga, minas gerais state, brazil. Annals of the New York Academy of Sciences 2006, 1078, 255-256.
51.	Garcia, M.N.; O'Day, S.; Fisher-Hoch, S.; Gorchakov, R.; Patino, R.; Arroyo, T.P.F.; Laing, S.T.; Lopez, J.E.; Ingber, A.; Jones, K.M., et al. One health interactions of chagas disease vectors, canid hosts, and human residents along the texas-mexico border. PLoS Neglected Tropical Diseases 2016, 10, p.e0005074.
52.	Gautam, R.; Srinath, I.; Clavijo, A.; Szonyi, B.; Bani-Yaghoub, M.; Park, S.; Ivanek, R. Identifying areas of high risk of human exposure to coccidioidomycosis in texas using serology data from dogs. Zoonoses and Public Health 2013, 60, 174-181.
53.	Gil, H.; Cano, L.; de Lucio, A.; Bailo, B.; de Mingo, M.H.; Cardona, G.A.; Fernandez-Basterra, J.A.; Aramburu-Aguirre, J.; Lopez-Molina, N.; Carmena, D. Detection and molecular diversity of giardia duodenalis and cryptosporidium spp. In sheltered dogs and cats in northern spain. Infection Genetics and Evolution 2017, 50, 62-69.
54.	Godsey, M.S., Jr.; Amoo, F.; Yuill, T.M.; DeFoliart, G.R. California serogroup virus infections in wisconsin domestic animals. American Journal of Tropical Medicine and Hygiene 1988, 39, 409-416.
55.	Gomez-Morales, M.A.; Selmi, M.; Ludovisi, A.; Amati, M.; Fiorentino, E.; Breviglieri, L.; Poglayen, G.; Pozio, E. Hunting dogs as sentinel animals for monitoring infections with trichinella spp. In wildlife. Parasites & Vectors 2016, 9, 154.
56.	Goossens, H.A.T.; Bogaard, A.E.v.d.; Nohlmans, M.K.E. Dogs as sentinels for human lyme borreliosis in the netherlands. Journal of Clinical Microbiology 2001, 39, 844-848.
57.	Grayzel, S.E.; Martinez-Lopez, B.; Sykes, J.E. Risk factors and spatial distribution of canine coccidioidomycosis in california, 2005-2013. Transboundary and Emerging Diseases 2017, 64, 1110-1119.
58.	Gurtler, R.E.; Ceballos, L.A.; Ordonez-Krasnowski, P.; Lanati, L.A.; Stariolo, R.; Kitron, U. Strong host-feeding preferences of the vector triatoma infestans modified by vector density: Implications for the epidemiology of chagas disease. PLoS Neglected Tropical Diseases 2009, 3, p.e447.
59.	Gurtler, R.E.; Wisnivesky-Colli, C.; Solarz, N.D.; Lauricella, M.; Bujas, M.A. Dynamics of transmission of trypanosoma cruzi in a rural area of argentina: Ii. Household infection patterns among children and dogs relative to the density of infected triatoma infestans. Bulletin of the Pan American Health Organization 1987, 21, 280-292.
60.	Hamer, S.A.; Tsao, J.I.; Walker, E.D.; Mansfield, L.S.; Foster, E.S.; Hickling, G.J. Use of tick surveys and serosurveys to evaluate pet dogs as a sentinel species for emerging lyme disease. American Journal of Veterinary Research 2009, 70, 49-56.
61.	Harrus, S.; Lior, Y.; Ephros, M.; Grisaru-Soen, G.; Keysary, A.; Strenger, C.; Jongejan, F.; Waner, T.; Baneth, G. Rickettsia conorii in humans and dogs: A seroepidemiologic survey of two rural villages in israel. American Journal of Tropical Medicine and Hygiene 2007, 77, 133-135.
62.	Havas, K.A.; Burkman, K. A comparison of the serological evidence of coxiella burnetii exposure between military working dogs and feral canines in iraq. Military Medicine 2011, 176, 1101-1103.
63.	Henn, J.B.; Gabriel, M.W.; Kasten, R.W.; Brown, R.N.; Theis, J.H.; Foley, J.E.; Chomel, B.B. Gray foxes (urocyon cinereoargenteus) as a potential reservoir of a bartonella clarridgeiae-like bacterium and domestic dogs as part of a sentinel system for surveillance of zoonotic arthropod-borne pathogens in northern california. Journal of Clinical Microbiology 2007, 45, 2411-2418.
64.	Herrero, C.; Pelaz, C.; Alvar, J.; Molina, R.; Vazquez, J.; Anda, P.; Casal, J.; Martin-Bourgon, C. Evidence of the presence of spotted fever group rickettsiae in dogs and dog ticks of the central provinces in spain. European Journal of Epidemiology 1992, 8, 575-579.
65.	Hidalgo, M.; Vesga, J.F.; Lizarazo, D.; Valbuena, G. A survey of antibodies against rickettsia rickettsii and ehrlichia chafeensis in domestic animals from a rural area of colombia. American Journal of Tropical Medicine and Hygiene 2009, 80, 1029-1030.
66.	Hilbink, F.; Penrose, M.; Kovacova, E.; Kazar, J. Q fever is absent from new zealand. International Journal of Epidemiology 1993, 22, 945-949.
67.	Hornok, S.; Denes, B.; Meli, M.L.; Tanczos, B.; Fekete, L.; Gyuranecz, M.; de la Fuente, J.; de Mera, I.G.F.; Farkas, R.; Hofmann-Lehmann, R. Non-pet dogs as sentinels and potential synanthropic reservoirs of tick-borne and zoonotic bacteria. Veterinary Microbiology 2013, 167, 700-703.
68.	Irwin, P.J.; Robertson, I.D.; Westman, M.E.; Perkins, M.; Straubinger, R.K. Searching for lyme borreliosis in australia: Results of a canine sentinel study. Parasites & Vectors 2017, 10, 114.
69.	Jaimes-Duenez, J.; Triana-Chavez, O.; Cantillo-Barraza, O.; Hernandez, C.; Ramirez, J.D.; Gongora-Orjuela, A. Molecular and serological detection of trypanosoma cruzi in dogs (canis lupus familiaris) suggests potential transmission risk in areas of recent acute chagas disease outbreaks in colombia. Preventive Veterinary Medicine 2017, 141, 1-6.
70.	Kelly, P.J.; Mason, P.R. Tick-bite fever in zimbabwe - survey of antibodies to rickettsia-conorii in man and dogs, and of rickettsia-like organisms in dog ticks. South African Medical Journal 1991, 80, 233-236.
71.	Kile, J.C.; Panella, N.A.; Komar, N.; Chow, C.C.; MacNeil, A.; Robbins, B.; Bunning, M.L. Serologic survey of cats and dogs during an epidemic of west nile virus infection in humans. Journal of the American Veterinary Medical Association 2005, 226, 1349-1353.
72.	Kilonzo, B.S.; Gisakanyi, N.D.; Sabuni, C.A. Involvement of dogs in plague epidemiology in tanzania - serological observations in domestic-animals in lushoto district. Scandinavian Journal of Infectious Diseases 1993, 25, 503-506.
73.	Koizumi, N.; Muto, M.M.; Akachi, S.; Okano, S.; Yamamoto, S.; Horikawa, K.; Harada, S.; Funatsumaru, S.; Ohnishi, M. Molecular and serological investigation of leptospira and leptospirosis in dogs in japan. Journal of Medical Microbiology 2013, 62, 630-636.
74.	Komar, N.; Panella, N.A.; Boyce, E. Exposure of domestic mammals to west nile virus during an outbreak of human encephalitis, new york city, 1999. (west nile virus). Emerging Infectious Diseases 2001, 7, 736-738.
75.	Krimer, P.M.; Phillips, K.M.; Miller, D.M.; Sanchez, S. Panniculitis attributable to mycobacterium goodii in an immunocompetent dog in georgia. Javma-Journal of the American Veterinary Medical Association 2010, 237, 1056-1059.
76.	Labruna, M.B.; Horta, M.C.; Aguiar, D.M.; Cavalcante, G.T.; Pinter, A.; Gennari, S.M.; Camargo, L.M.A. Prevalence of rickettsia infection in dogs from the urban and rural areas of monte negro municipality, western amazon, brazil. Vector Borne and Zoonotic Diseases 2007, 7, 249-255.
77.	Lan, D.; Ji, W.; Yu, D.; Chu, J.; Wang, C.; Yang, Z.; Hua, X. Serological evidence of west nile virus in dogs and cats in china. Archives of Virology 2011, 156, 893-895.
78.	Langoni, H.; Matteucci, G.; Medici, B.; Camossi, L.G.; Richini-Pereira, V.B.; da Silva, R.C. Detection and molecular analysis of toxoplasma gondii and neospora caninum from dogs with neurological disorders. Revista Da Sociedade Brasileira De Medicina Tropical 2012, 45, 365-368.
79.	Lauzi, S.; Maia, J.P.; Epis, S.; Marcos, R.; Pereira, C.; Luzzago, C.; Santos, M.; Puente-Payo, P.; Giordano, A.; Pajoro, M., et al. Molecular detection of anaplasma platys, ehrlichia canis, hepatozoon canis and rickettsia monacensis in dogs from maio island of cape verde archipelago. Ticks and Tick-Borne Diseases 2016, 7, 964-969.
80.	Lembo, T.; Hampson, K.; Auty, H.; Beesley, C.A.; Bessell, P.; Packer, C.; Halliday, J.; Fyumagwa, R.; Hoare, R.; Ernest, E., et al. Serologic surveillance of anthrax in the serengeti ecosystem, tanzania, 1996-2009. Emerging Infectious Diseases 2011, 17, 387-394.
81.	Lemos, E.R.S.d.; Machado, R.D.; Coura, J.R.; Guimaraes, M.A.A.M.; Chagas, N. Epidemiological aspects of the brazilian spotted fever: Serological survey of dogs and horses in an endemic area in the state of sao paulo, brazil. Revista do Instituto de Medicina Tropical de Sao Paulo 1996, 38, 427-430.
82.	Levanov, L.; Vera, C.P.; Vapalahti, O. Prevalence estimation of tick-borne encephalitis virus (tbev) antibodies in dogs from finland using novel dog anti-tbev igg mab-capture and igg immunofluorescence assays based on recombinant tbev subviral particles. Ticks and Tick borne Diseases 2016, 7, 979-982.
83.	Levy, J.K.; Crawford, P.C.; Lappin, M.R.; Dubovi, E.J.; Levy, M.G.; Alleman, R.; Tucker, S.J.; Clifford, E.L. Infectious diseases of dogs and cats on isabela island, galapagos. Journal of Veterinary Internal Medicine 2008, 22, 60-65.
84.	Li, B.; Guo, Y.; Guo, Z.B.; Liang, Y.; Zhu, Z.W.; Zhou, Q.; Yan, Y.F.; Song, Z.Z.; Yang, R.F. Serologic survey of the sentinel animals for plague surveillance and screening for complementary diagnostic markers to f1 antigen by protein microarray. American Journal of Tropical Medicine and Hygiene 2008, 79, 799-802.
85.	Lindenmayer, J.M.; Marshall, D.; Onderdonk, A.B. Dogs as sentinels for lyme-disease in massachusetts. American Journal of Public Health 1991, 81, 1448-1455.
86.	Lindhe, K.E.S.; Meldgaard, D.S.; Jensen, P.M.; Houser, G.A.; Berendt, M. Prevalence of tick-borne encephalitis virus antibodies in dogs from denmark. Acta Veterinaria Scandinavica 2009, 51, 56.
87.	Lledo, L.; Gegundez, M.I.; Serrano, J.L.; Saz, J.V.; Beltran, M. A sero-epidemiological study of rickettsia typhi infection in dogs from soria province, central spain. Annals of Tropical Medicine and Parasitology 2003, 97, 861-864.
88.	Londono, A.F.; Acevedo-Gutierrez, L.Y.; Marin, D.; Contreras, V.; Diaz, F.J.; Valbuena, G.; Labruna, M.B.; Hidalgo, M.; Arboleda, M.; Mattar, S., et al. Wild and domestic animals likely involved in rickettsial endemic zones of northwestern colombia. Ticks and Tick-Borne Diseases 2017, 8, 887-894.
89.	Lunn, J.A.; Lee, R.; Smaller, J.; MacKay, B.M.; King, T.; Hunt, G.B.; Martin, P.; Krockenberger, M.B.; Spielman, D.; Malik, R. Twenty two cases of canine neural angiostronglyosis in eastern australia (2002-2005) and a review of the literature. Parasites and Vectors 2012, 5, 70.
90.	Magnarelli, L.A.; Anderson, J.F.; Philip, R.N.; Burgdorfer, W. Antibodies to spotted fever-group rickettsiae in dogs and prevalence of infected ticks in southern connecticut. American Journal of Veterinary Research 1982, 43, 656-659.
91.	Maia, C.; Alwassouf, S.; Cristovao, J.M.; Ayhan, N.; Pereira, A.; Charrel, R.N.; Campino, L. Serological association between leishmania infantum and sand fly fever sicilian (but not toscana) virus in sheltered dogs from southern portugal. Parasites & Vectors 2017, 10, 92.
92.	Mannelli, A.; Cerri, D.; Buffrini, L.; Rossi, S.; Rosati, S.; Arata, T.; Innocent, M.; Grignolo, M.C.; Bianchi, G.; Iori, A., et al. Low risk of lyme borreliosis in a protected area on the tyrrhenian coast, in central italy. European Journal of Epidemiology 1999, 15, 371-377.
93.	Matete, G.O. Occurrence, clinical manifestation and the epidemiological implications of naturally occurring canine trypanosomosis in western kenya. Onderstepoort Journal of Veterinary Research 2003, 70, 317-323.
94.	McQuiston, J.H.; Guerra, M.A.; Watts, M.R.; Lawaczeck, E.; Levy, C.; Nicholson, W.L.; Adjemian, J.; Swerdlow, D.L. Evidence of exposure to spotted fever group rickettsiae among arizona dogs outside a previously documented outbreak area. Zoonoses and Public Health 2011, 58, 85-92.
95.	Mead, P.; Goel, R.; Kugeler, K. Canine serology as adjunct to human lyme disease surveillance. Emerging infectious diseases 2011, 17, 1710.
96.	Meireles, L.R.; Galisteo Junior, A.J.; Pompeu, E.; Andrade Junior, H.F. Toxoplasma gondii spreading in an urban area evaluated by seroprevalence in free-living cats and dogs. Tropical Medicine and International Health 2004, 9, 876-881.
97.	Merino, F.J.; Serrano, J.L.; Saz, J.V.; Nebreda, T.; Gegundez, M.; Beltran, M. Epidemiological characteristics of dogs with lyme borreliosis in the province of soria (spain). European Journal of Epidemiology 2000, 16, 97-100.
98.	Milagres, B.S.; Padilha, A.F.; Barcelos, R.M.; Gomes, G.G.; Montandon, C.E.; Pena, D.C.H.; Bastos, F.A.N.; Silveira, I.; Pacheco, R.; Labruna, M.B., et al. Rickettsia in synanthropic and domestic animals and their hosts from two areas of low endemicity for brazilian spotted fever in the eastern region of minas gerais, brazil. American Journal of Tropical Medicine and Hygiene 2010, 83, 1305-1307.
99.	Millán, J.; Chirife, A.D.; Kalema-Zikusoka, G.; Cabezón, O.; Muro, J.; Marco, I.; Cliquet, F.; León-Vizcaíno, L.; Wasniewski, M.; Almería, S. Serosurvey of dogs for human, livestock, and wildlife pathogens, uganda. Emerging infectious diseases 2013, 19, 680.
100.	Miro, G.; Montoya, A.; Mateo, M.; Alonso, A.; Garcia, S.; Garcia, A.; Caballero, M.J.; Molina, R. A leishmaniosis surveillance system among stray dogs in the region of madrid: Ten years of serodiagnosis (1996-2006). Parasitology Research 2007, 101, 253-257.
101.	Miro, G.; Muller, A.; Montoya, A.; Checa, R.; Marino, V.; Marino, E.; Fuster, F.; Escacena, C.; Descalzo, M.A.; Galvez, R. Epidemiological role of dogs since the human leishmaniosis outbreak in madrid. Parasites & Vectors 2017, 10, 209.
102.	Molina Barrios, R.M.; Lucero Cebreros, J.R.C.C.A.; Gastelum Castaneda, A.; Luevano Adame, J.d.l.L. Detection of antibodies against borrelia burgdorferi in dogs from southern of sonora. 3rd Biotechnology Summit 2016, Ciudad Obregón, Sonora, Mexico, 24-28 October 2016 2016, 12-15.
103.	Moore, G.E.; Guptill, L.F.; Glickman, N.W.; Caldanaro, R.J.; Aucoin, D.; Glickman, L.T. Canine leptospirosis, united states, 2002–2004. Emerging infectious diseases 2006, 12, 501.
104.	Moreira-Soto, A.; Carranza, M.V.; Taylor, L.; Calderon-Arguedas, O.; Hun, L.; Troyo, A. Exposure of dogs to spotted fever group rickettsiae in urban sites associated with human rickettsioses in costa rica. Ticks and Tick-Borne Diseases 2016, 7, 748-753.
105.	Morikawa, V.M.; Bier, D.; Pellizzaro, M.; Ullmann, L.S.; Paploski, I.A.D.; Kikuti, M.; Langoni, H.; Biondo, A.W.; Molento, M.B. Seroprevalence and seroincidence of leptospira infection in dogs during a one-year period in an endemic urban area in southern brazil. Revista Da Sociedade Brasileira De Medicina Tropical 2015, 48, 50-55.
106.	Nichols, J.B. Evaluation of military sentry dogs as a sentinel system to venezuelan equine encephalitis. Military Medicine 1975, 140:p710-711, Secondary journal source: Veterinary Medicine & Small Animal Clinician (1976) 1971(1972) p1127.
107.	Olson, P.E.; Kallen, A.J.; Bjorneby, J.M.; Creek, J.G. Canines as sentinels for lyme disease in san diego county, california. Journal of Veterinary Diagnostic Investigation 2000, 12, 126-129.
108.	Ortega-Pacheco, A.; Guzman-Marin, E.; Acosta-Viana, K.Y.; Vado-Solis, I.; Jimenez-Delgadillo, B.; Cardenas-Marrufo, M.; Perez-Osorio, C.; Puerto-Solis, M.; Jimenez-Coello, M. Serological survey of leptospira interrogans, toxoplasma gondii and trypanosoma cruzi in free roaming domestic dogs and cats from a marginated rural area of yucatan mexico. Veterinary Medicine and Science 2017, 3, 40-47.
109.	Ortiz, S.; Ceballos, M.J.; Gonzalez, C.R.; Reyes, C.; Gomez, V.; Garcia, A.; Solari, A. Trypanosoma cruzi diversity in infected dogs from areas of the north coast of chile. Veterinary Parasitology: Regional Studies and Reports 2016, 5, 42-47.
110.	Otomura, F.H.; Truppel, J.H.; Moraes Filho, J.; Labruna, M.B.; Rossoni, D.F.; Massafera, R.; Soccol, V.T.; Teodoro, U. Probability of occurrence of the brazilian spotted fever in northeast of parana state, brazil. Brazilian Journal of Veterinary Parasitology 2016, 25, 394-400.
111.	Pejchalova, K.; Zakovska, A.; Fucik, K.; Schanilec, P. Serological confirmation of borrelia burgdorferi infection in dogs in the czech republic. Veterinary Research Communications 2006, 30, 231-238.
112.	Pinter, A.; Horta, M.C.; Pacheco, R.C.; Moraes Filho, J.; Labruna, M.B. Serosurvey of rickettsia spp. In dogs and humans from an endemic area for brazilian spotted fever in the state of sao paulo, brazil. Cadernos de Saude Publica 2008, 24, 247-252.
113.	Potkonjak, A.; Petrovic, T.; Ristanovic, E.; Lalic, I.; Vracar, V.; Savic, S.; Turkulov, V.; Canak, G.; Milosevic, V.; Vidanovic, D., et al. Molecular detection and serological evidence of tick-borne encephalitis virus in serbia. Vector-Borne and Zoonotic Diseases 2017, 17, 813-820.
114.	Pryor, W.H.; Irving, G.S.; Kundin, W.D.; Taylor, G.D.; Ziegler, R.F.; Dixon, D.F., Jr.; Hinkle, D.K. A serologic survey of military personnel and dogs in thailand and south vietnam for antibodies to arboviruses, rickettsia tsutsugamushi, and pseudomonas pseudomallei. American Journal of Veterinary Research 1972, 33, 2091-2095.
115.	Quintero, V.J.; Paternina, T.L.; Uribe, Y.A.; Muskus, C.; Hidalgo, M.; Gil, J.; Cienfuegos, G.A.; Osorio, Q.L.; Rojas, A.C. Eco-epidemiological analysis of rickettsial seropositivity in rural areas of colombia: A multilevel approach. PLoS Negl Trop Dis 2017, 11, e0005892.
116.	Rand, P.W.; Lacombe, E.H.; Smith, R.P., Jr.; Gensheimer, K.; Dennis, D.T. Low seroprevalence of human lyme disease near a focus of high entomologic risk. American Journal of Tropical Medicine and Hygiene 1996, 55, 160-164.
117.	Resnick, M.P.; Grunenwald, P.; Blackmar, D.; Hailey, C.; Bueno, R.; Murray, K.O. Juvenile dogs as potential sentinels for west nile virus surveillance. Zoonoses and public health 2008, 55, 443-447.
118.	Rocheleau, J.P.; Michel, P.; Lindsay, L.R.; Drebot, M.; Dibernardo, A.; Ogden, N.H.; Fortin, A.; Arsenault, J. Emerging arboviruses in quebec, canada: Assessing public health risk by serology in humans, horses and pet dogs. Epidemiology and Infection 2017, 145, 2940-2948.
119.	Roelandt, S.; Heyman, P.; Filette, M.d.; Vene, S.; Stede, Y.v.d.; Caij, A.B.; Tavernier, P.; Dobly, A.; Bosschere, H.d.; Vyt, P., et al. Tick-borne encephalitis virus seropositive dog detected in belgium: Screening of the canine population as sentinels for public health. Vector Borne and Zoonotic Diseases 2011, 11, 1371-1376.
120.	Roque, A.L.R.; Xavier, S.C.C.; Gerhardt, M.; Silva, M.F.O.; Lima, V.S.; D'Andrea, P.S.; Jansen, A.M. Trypanosoma cruzi among wild and domestic mammals in different areas of the abaetetuba municipality (para state, brazil), an endemic chagas disease transmission area. Veterinary Parasitology 2013, 193, 71-77.
121.	Sakhria, S.; Alwassouf, S.; Fares, W.; Bichaud, L.; Dachraoui, K.; Alkan, C.; Zoghlami, Z.; de Lamballerie, X.; Zhioua, E.; Charrel, R.N. Presence of sandfly-borne phleboviruses of two antigenic complexes (sandfly fever naples virus and sandfly fever sicilian virus) in two different bio-geographical regions of tunisia demonstrated by a microneutralisation-based seroprevalence study in dogs. Parasites & Vectors 2014, 7, 476.
122.	Salb, A.L.; Barkema, H.W.; Elkin, B.T.; Thompson, R.C.A.; Whiteside, D.P.; Black, S.R.; Dubey, J.R.; Kutz, S.J. Dogs as sources and sentinels of parasites in humans and wildlife, northern canada. Emerging Infectious Diseases 2008, 14, 60-63.
123.	Schule, C.; Rehbein, S.; Shukullari, E.; Rapti, D.; Reese, S.; Silaghi, C. Police dogs from albania as indicators of exposure risk to toxoplasma gondii, neospora caninum and vector-borne pathogens of zoonotic and veterinary concern. Veterinary Parasitology: Regional Studies and Reports 2015, 1, 35-46.
124.	Schuller, S.; Arent, Z.J.; Gilmore, C.; Nally, J. Prevalence of antileptospiral serum antibodies in dogs in ireland. Veterinary Record 2015, 177, 126.
125.	Schurer, J.M.; Hill, J.E.; Fernando, C.; Jenkins, E.J. Sentinel surveillance for zoonotic parasites in companion animals in indigenous communities of saskatchewan. American Journal of Tropical Medicine and Hygiene 2012, 87, 495-498.
126.	Schurer, J.M.; Ndao, M.; Quewezance, H.; Elmore, S.A.; Jenkins, E.J. People, pets, and parasites: One health surveillance in southeastern saskatchewan. American Journal of Tropical Medicine and Hygiene 2014, 90, 1184-1190.
127.	Shadomy, S.V.; Waring, S.C.; Chappell, C.L. Combined use of enzyme-linked immunosorbent assay and flow cytometry to detect antibodies to trypanosoma cruzi in domestic canines in texas. Clinical and Diagnostic Laboratory Immunology 2004, 11, 313-319.
128.	Shang, L.; Peng, W.; Jin, H.; Xu, D.; Zhong, N.; Wang, W.; Wu, Y.; Liu, Q. The prevalence of canine leishmania infantum infection in sichuan province, southwestern china detected by real time pcr. Parasites and Vectors 2011, 4, 173.
129.	Shapiro, A.J.; Brown, G.; Norris, J.M.; Bosward, K.L.; Marriot, D.J.; Nandhakumar, B.; Breitschwerdt, E.B.; Malik, R. Vector-borne and zoonotic diseases of dogs in north-west new south wales and the northern territory, australia. BMC Veterinary Research 2017, 13, 238.
130.	Shimoda, H.; Ohno, Y.; Mochizuki, M.; Iwata, H.; Okuda, M.; Maeda, K. Dogs as sentinels for human infection with japanese encephalitis virus. Emerging Infectious Diseases 2010, 16, 1137-1139.
131.	Silva, J.R.d.; Maciel, B.M.; Santos, L.K.N.d.S.S.; Carvalho, F.S.; Rocha, D.d.S.; Lopes, C.W.G.; Albuquerque, G.R. Isolation and genotyping of toxoplasma gondii in brazilian dogs. Korean Journal of Parasitology 2017, 55, 239-246.
132.	Smith, F.D.; Ballantyne, R.; Morgan, E.R.; Wall, R. Estimating lyme disease risk using pet dogs as sentinels. Comparative Immunology Microbiology and Infectious Diseases 2012, 35, 163-167.
133.	Starkey, L.A.; Barrett, A.W.; Chandrashekar, R.; Stillman, B.A.; Tyrrell, P.; Thatcher, B.; Beall, M.J.; Gruntmeir, J.M.; Meinkoth, J.H.; Little, S.E. Development of antibodies to and pcr detection of ehrlichia spp. In dogs following natural tick exposure. Veterinary Microbiology 2014, 173, 379-384.
134.	Takashima, I. Epidemiology of tick-borne encephalitis in japan. Comparative Immunology Microbiology and Infectious Diseases 1998, 21, 81-90.
135.	Tenney, T.D.; Curtis-Robles, R.; Snowden, K.F.; Hamer, S.A. Shelter dogs as sentinels for trypanosoma cruzi transmission across texas, usa. Emerging Infectious Diseases 2014, 20, 1323-1326.
136.	Tomassone, L.; Conte, V.; Parrilla, G.; Meneghi, D.d. Rickettsia infection in dogs and rickettsia parkeri in amblyomma tigrinum ticks, cochabamba department, bolivia. Vector Borne and Zoonotic Diseases 2010, 10, 953-958.
137.	Vieira, T.S.W.J.; Vieira, R.F.d.C.; Nascimento, D.A.G.d.; Tamekuni, K.; Toledo, R.d.S.; Ramaswamy, C.; Marcondes, M.; Biondo, A.W.; Vidotto, O. Serosurvey of tick-borne pathogens in dogs from urban and rural areas from parana state, brazil. Revista Brasileira de Parasitologia Veterinaria 2013, 22, 104-109.
138.	Watier-Grillot, S.; Vallee, I.; Lacour, S.A.; Cana, A.; Davoust, B.; Marie, J.L. Strayed dogs sentinels of trichinella britovi infection in kosovo. Parasite-Journal De La Societe Francaise De Parasitologie 2011, 18, 281-283.
139.	Willeberg, P.; Ruppanner, R.; Behymer, D.E.; Haghighi, S.; Kaneko, J.J.; Franti, C.E. Environmental exposure to coxiella burnetii: A sero-epidemiologic survey among domestic animals. American Journal of Epidemiology 1980, 111, 437-443.
140.	Xavier, S.C.D.; Roque, A.L.R.; Lima, V.D.; Monteiro, K.J.L.; Otaviano, J.C.R.; da Silva, L.; Jansen, A.M. Lower richness of small wild mammal species and chagas disease risk. PLoS Neglected Tropical Diseases 2012, 6, p.e1647.
141.	Yan, C.; Fu, L.L.; Yue, C.L.; Tang, R.X.; Liu, Y.S.; Lv, L.; Shi, N.; Zeng, P.; Zhang, P.; Wang, D.H., et al. Stray dogs as indicators of toxoplasma gondii distributed in the environment: The first report across an urban-rural gradient in china. Parasites & Vectors 2012, 5, 5.
142.	Zhao, S.S.; Pulati, Y.; Yin, X.P.; Li, W.; Wang, B.J.; Yang, K.; Chen, C.F.; Wang, Y.Z. Wildlife plague surveillance near the china-kazakhstan border: 2012-2015. Transboundary and Emerging Diseases 2017, 64, E48-E51.
