# Supplementary material for: Dogs (Canis familiaris) as Sentinels for Human Infectious Disease and Application to Canadian Populations: A Systematic Review
Source: Vet Sci. 2018 Sep 21;5(4):83. doi: 10.3390/vetsci5040083 (PMC6313866; doi:10.3390/vetsci5040083)
Supplement: Supplementary file 1 [file vetsci-05-00083-s001.zip › S2 Example of completed data capture form.rtf]

GENERAL INFORMATION		
Reference	GOOSSENS, H. A. T., et al. (2001) Dogs as sentinels for human Lyme borreliosis in The Netherlands. Journal of Clinical Microbiology, 39 (3), 844-848.	
Peer reviewed?	Yes	
Abstract	Serum samples from hunters (n = 440), their hunting dogs (n = 448), and hunters without dog ownership (n = 53) were collected in The Netherlands at hunting dog trials and were tested for antibodies against Borrelia burgdorferi by a whole-cell enzyme-linked immunosorbent assay. Additionally, 75 healthy pet dogs were tested. The results of this study indicate that the seroprevalence among hunting dogs (18%) was of the same order as the seroprevalence among pet dogs (17%) and hunters (15%). The seropositivity of a hunting dog was not a significant indicator of increased risk of Lyme borreliosis for its owner. No significant rise in seroprevalence was found in dogs older than 24 months. This indicated that seropositivity after an infection with B. burgdorferi in dogs is rather short, approximately 1 year. In humans this is considerably longer but is also not lifelong. Therefore, the incidence of B. burgdorferi infections among dogs was greater than that among hunters, despite a similar prevalence of seropositivity among hunters and their hunting dogs. Because no positive correlation was observed between the seropositivity of a hunter and the seropositivity of the hunter's dog, direct transfer of ticks between dog and hunter does not seem important and owning a dog should not be considered a risk factor for Lyme borreliosis.	
CONCLUSION AS TO USE OF DOGS AS SENTINELS	Demonstrating use of dogs as sentinels	
METHODOLOGY		
Type of study	Sero-prevalence of hunting dogs and owners	
Type of intervention / method	ELISA	
SPATIAL-TEMPORAL INFORMATION		
Year	1989	
Continent 	Europe	
Country	The Netherlands	
Region	Not specified	
DISEASE INFORMATION		
Disease	Borrelia burgdorferi (Lyme borreliosis)	
Type of disease	Bacterial, tick-borne	
POPULATIONS		
Canid population	440 hunting dogs	
Human population	448 hunters (paired owners of the dogs)	
RELEVANCE TO CANADA		
Paper from Canada?	No	
Disease found in Canada?	Yes	
Relevant populations found in Canada?	Yes	
Disease found in the USA?	Yes	
Possible route of transmission to Canada?	Already present	
Other relevant information	Surprisingly, although it was supposed that hunting dogs have an increased risk of tick infestation compared to the risk for other dogs, no significant differences in seroprevalences between the hunting dog and the pet dog populations were found. These findings are in contrast to the seroprevalence among hunting dogs (40%) in the Slovak Republic (43), which was significantly higher than the seroprevalence among service dogs (12%).
The use of dog sera to detect and quantify the risk of Lyme disease for humans in a certain region is more sensitive than the use of reports of incident human clinical cases but is not more sensitive than the use of seroprevalence in humans. The use of dog sera, however, has the advantage that the seroprevalence among dogs is more likely to reflect the actual environmental risk of Lyme disease because of the short half life of canine antibodies against B. burgdorferi.
Only 12% of the seropositive hunters had hunting dogs which were also seropositive.	
